# Spatiotemporal heterogeneity of LMOD1 expression summarizes two modes of cell communication in colorectal cancer

**DOI:** 10.1186/s12967-024-05369-3

**Published:** 2024-06-07

**Authors:** Jie-pin Li, Yuan-jie Liu, Yang Li, Yi Yin, Qian-wen Ye, Zhi-hua Lu, Yu-wei Dong, Jin-yong Zhou, Xi Zou, Yu-gen Chen

**Affiliations:** 1 Jiangsu Province Hospital of Chinese Medicine, The Affiliated Hospital of Nanjing University of Chinese Medicine, Hanzhong Road No.155, Nanjing, 210029 Jiangsu China; 2 Jiangsu Province Key Laboratory of Tumor Systems Biology and Chinese Medicine, Nanjing, 210029 Jiangsu China; 3 Nanjing University of Chinese Medicine, Nanjing, 210029 Jiangsu China; 4 Central Laboratory, Affiliated Hospital of Nanjing University of Chinese Medicine, Jiangsu Province Hospital of Chinese Medicine, Nanjing, 210029 Jiangsu China; 5 Institute of Chinese & Western Medicine and Oncology Clinical Research, Nanjing, 210029 Jiangsu China; 6 Jiangsu Collaborative Innovation Center of Traditional Chinese Medicine in Prevention and Treatment of Tumor, Nanjing, 210029 Jiangsu China

**Keywords:** Colorectal cancer, Tight junctions, Gap junctions, Epithelial cells, Fibroblasts, LMOD1

## Abstract

Cellular communication (CC) influences tumor development by mediating intercellular junctions between cells. However, the role and underlying mechanisms of CC in malignant transformation remain unknown. Here, we investigated the spatiotemporal heterogeneity of CC molecular expression during malignant transformation. It was found that although both tight junctions (TJs) and gap junctions (GJs) were involved in maintaining the tumor microenvironment (TME), they exhibited opposite characteristics. Mechanistically, for epithelial cells (parenchymal component), the expression of TJ molecules consistently decreased during normal-cancer transformation and is a potential oncogenic factor. For fibroblasts (mesenchymal component), the expression of GJs consistently increased during normal-cancer transformation and is a potential oncogenic factor. In addition, the molecular profiles of TJs and GJs were used to stratify colorectal cancer (CRC) patients, where subtypes characterized by high GJ levels and low TJ levels exhibited enhanced mesenchymal signals. Importantly, we propose that leiomodin 1 (LMOD1) is biphasic, with features of both TJs and GJs. LMOD1 not only promotes the activation of cancer-associated fibroblasts (CAFs) but also inhibits the Epithelial–mesenchymal transition (EMT) program in cancer cells. In conclusion, these findings demonstrate the molecular heterogeneity of CC and provide new insights into further understanding of TME heterogeneity.

**Graphical Abstract:**

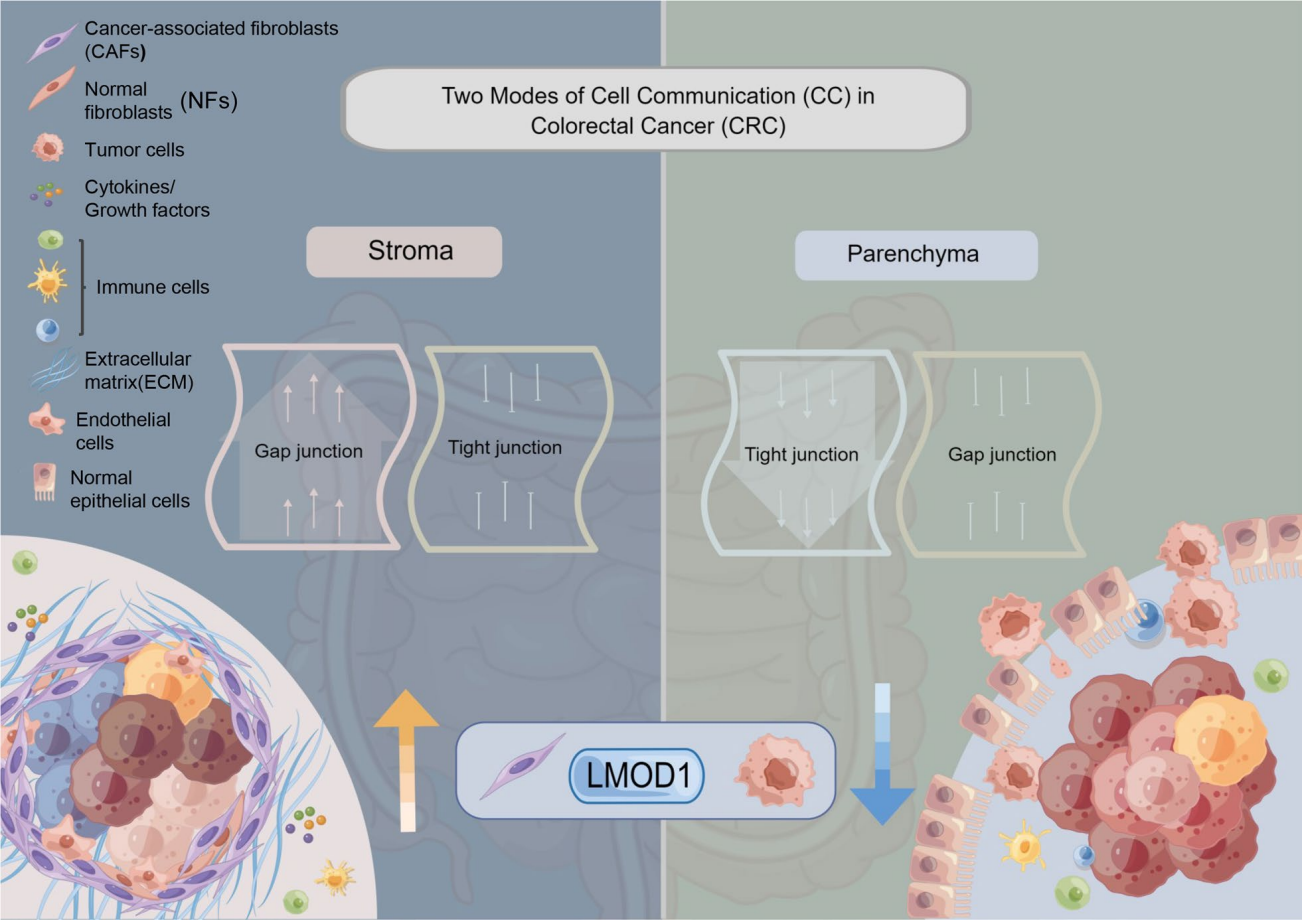

**Supplementary Information:**

The online version contains supplementary material available at 10.1186/s12967-024-05369-3.

## Highlights

1. Tight junctions are involved in malignant transformation of intestinal epithelial cells.
2. Gap junctions are involved in malignant transformation of fibroblasts.
3. LMOD1 is identified to resembles tight junctions in the epithelium and is similar to gap junctions in the stroma.
4. LMOD1 regulates FGF1 expression in the stroma to promote tumorigenesis and is regulated by AKAP12 in the epithelium to inhibit tumorigenesis.
5. LMOD1 is associated with low immunotherapy response rates in colorectal cancer patients.

**Supplementary Information:**

The online version contains supplementary material available at 10.1186/s12967-024-05369-3.

## Introduction

Colorectal cancer (CRC) is the third most common cancer and the leading cause of death among gastrointestinal tumors worldwide [1]. Although chemoradiotherapy is an effective option for CRC, the overall prognosis for patients remains unsatisfactory [2]. Recently, the standardized implementation of tumor immunotherapy and targeted therapies, especially immune checkpoint blockades (ICBs), has greatly prolonged patient survival [3]. However, a significant proportion of patients do not respond to ICBs, which is often accompanied by various degrees of adverse effects [4, 5]. Considering the individualized significance of cancer treatment, there is a need for more effective and precise treatment protocols. It is well documented that cancer cells’ survival depends on the tumor microenvironment (TME), which is essentially involved in the response rate to immunotherapy, and the impressive heterogeneity of the TME poses a great difficulty in treatment [6, 7]. Patients with tumor-infiltrating lymphocytes (TILs) with difficulty in effectively infiltrating the tumor do not significantly respond to ICBs [8, 9].

Cellular communication (CC) is a pervasive biological mechanism by which cells are influenced by signaling pathways and physical parameters in their environment during development and disease [10, 11]. Recently, the association between CC and cancer has been well demonstrated [12–14]. It has been shown that weakening of tight junctions (TJs) is essential for cancer cells to turn on the epithelial–mesenchymal transition (EMT) program, whereas increased expression of claudin (CLDN) proteins decreases the cancer cell’s ability to invade and metastasize [15, 16]. Hulikova et al. demonstrated that tumor-stromal cell interactions influence cancer progression and fibroblasts help cancer cells maintain a favorable pH, which is necessary for cancer cells’ ability to proliferate and invade, through the formation of gap junctions (GJs) between them [17]. Understanding the mechanistic basis of intercellular communication during tumor progression is fundamental to inhibiting cancer cell metastasis.

The aforementioned studies involved only one or two molecules associated with CC, but the heterogeneity of TME is the result of multiple, deeply interrelated molecules. Currently, there is no comprehensive study on the tumor CC profile of TME, and the heterogeneity of CC molecules in tumors remains undetermined. It has been indicated that during tumor formation, TJ molecules are in an expression-deficient state, inhibiting most cancer cells from forming dense TJ structures, which may be related to genomic regulation and upstream molecular signaling [18, 19]. Therefore, analyzing the temporal and spatial characteristics of gene expression in tumors and understanding the correlation between various CC molecules and TME are important for determining the specific effects of CC on tumors.

The present study analyzed the gene expression heterogeneity of 47 CC molecules in The Cancer Genome Atlas (TCGA) pan-cancer database and compared normal and tumor tissues. The data indicated that genomic variants affected the expression of CC molecules. We first explained the spatiotemporal heterogeneity of CC molecules in tumor progression and linked CC molecules to the immune profile of the TME using transcriptomic information in TCGA-CRC. The results revealed that CC has a significant impact on the TME. We identified leiomodin 1 (LMOD1)—a potential regulator of the CC phenotype with biphasic features of both GJs and TJs. It was confirmed that LMOD1 is a factor in poor immune infiltration of CRC and can be used to develop new targeted drugs to improve immunotherapy response rates.

## Materials and methods

All the antibodies and reagents utilized in this study are listed in the Supplemental Material (Table S1). Antibody concentrations utilized were per the instructions provided by the relevant company or as indicated in the literature. Furthermore, all details regarding data analyses, participants’ inclusion/exclusion parameters, tables, figures, etc. are presented as supplementary data.

### Public datasets

A total of 620 CRC samples were acquired from the University of California Santa Cruz (UCSC) website [20] and included gene expression, clinical, and mutation data. TCGA-CRC gene expression profiles (Fragments Per Kilobase of transcript per Million mapped reads [FPKM] values) were transformed into Transcripts Per kilobase Million (TPM) using R software. R software was used to construct the data matrix for further analysis of gene expression data. In addition, some TCGA data analyses were performed using the GSCALite web tool [21].

The spatial transcriptome (ST) data were obtained from the Gene Expression Omnibu (GEO) database. The normal intestinal and CRC tissue ST data can be accessed through GEO under accession numbers GSE158328 [22] and GSE206552 [23]. The “SpatialDimplot” function from the “Seurat” package was used to obtain the position information of each gene.

The Genetic Perturbation dataset GSE147739 was retrieved from genetic perturbation similarity analysis database (GPSAdb) (gene: LOMD1; cell line: SW480) [24]. Relevant transcriptomic analyses were also completed based on this database.

### Survival and expression analyses

The “survival” package in R and the public dataset were utilized for mortality analysis. The assays comprised overall survival (OS), progression-free interval (PFI), and disease-specific survival (DSS). Furthermore, a paired t-test was carried out to elucidate the differences in TCGA-CRC among paired samples, and the data was visualized via the “ggplot2” package [25].

### Consensus clustering

Data on molecules of TJs and GJs from the Human Genome Organization (HUGO) portal are summarized in Table S2 [26]. Furthermore, clustering analysis was performed on the expression profiles of 47 molecules to identify CC phenotypes that are linked to CRC and stratify CRC patients. Moreover, the most ideal CRC-cohort clustering quantity was established via the consensus clustering algorithm, and then its stability was validated. All the analyses were performed while employing the R package “ConsensusClusterPlus” and were repeated 50 times with an 80% resampling rate [27].

### Screening of CRC-related differentially expressed genes (DEGs)

CRC-associated DEGs were identified using the “limma” package and ranked by absolute log_2_ fold change (FC) > 2 and P < 0.05. Significant DEGs were assessed in the subsequent analyses [28].

### Establishment of co-expression network

The “multiscale embedded gene co-expression network analysis (MEGENA)” package in R [29]—a recently established co-expression module analysis tool with new advantages for efficient gene association preservation and large-scale co-expression plane filtering axes formation—was utilized for co-expression network analysis. MEGENA analysis involved the formation of a fast planar filtered network (PFN), computational identification of specific PFN gene pairs, aggregation of established PFNs for multiscale clustering analysis (MCA), and other assessments. A readable document was generated for the acquired gene module-based largest co-expression network via Cytoscape for visualization and final assessment. The degree values were assessed for stratifying the module genes for the determination of possible hub genes.

### Immunologic evaluation

The “Cibersort” algorithm was utilized to predict TCGA-CRC sample immune cell infiltration [30]. With the help of the “ggplot2” R package, a box plot was established that indicated the immune cell abundance differences. Furthermore, the scores for Tumor immune dysfunction and exclusion (TIDE) were also investigated [31], which demonstrated the potential immune checkpoint (ICB) blockade response in CRC patients.

### Preparation of single cell suspensions from surgical specimen

Surgical specimens were assessed using single-cell RNA sequencing (scRNA-seq) to evaluate the molecular characteristics and cell populations of CRC progression after normal mucosa developed into adenoma and eventually malignant adenocarcinoma. Normal epithelium, adenoma, and adenocarcinoma were selected from the same patient. biological replicates were performed on three patients, and nine samples were included. The study protocol was approved by the ethics committee of Jiangsu Province Hospital of Chinese Medicine, and informed consent was obtained from clinicians and patients (2021NL-206–01). Chemotherapy and radiotherapy were not administered to the patients before surgery. A rapid intraoperative pathological examination was performed. All tissues were diagnosed by senior pathologists in the Department of Pathology. Each fresh specimen from surgery was subjected to subsequent protocols. The biopsy samples were sliced into small pieces using an Iris scissor, inoculated for 30 min in the digestion solution augmented with phosphate-buffered saline (PBS) at 37 °C and 800 rpm, and then incubated with collagenase III, trypsin, and deoxyribonuclease (DNase) at 37 °C for 1 h. After dilution of cell suspension with 4 mL of Dulbecco’s modified Eagles medium (DMEM), cells were passed via a 40-m cell mesh, spun for 5 min at 250*g*, and separated from the buoyant. Cells were then rinsed twice with phosphate-buffered saline (PBS). The cell pellet was resuspended using the solution of red blood cell lysis, incubated at 4 °C for 10 min, mixed with chilled PBS (10 mL), and centrifuged for 10 min at 250*g*. Then, the pellet was re-dissolved in PBS (5 mL) without magnesium or calcium and bovine serum albumin (BSA; 0.04% w/v). The suspension (10 µL) was quantified using a hemocytometer under the inverted microscope using trypan blue to assess the number of live cells.

### scRNA data processing

The 10× Genomics sequencing data were aligned and measured against a human reference genome (hg19) via the CellRanger package (version 3.1) [32]. Cells with A library size < 200, mitochondrial transcript ratio > 0.4, and gene expression < 3 were removed. The remaining gene expression matrix of 20,262 cells was normalized and then adjusted by regressing the total cellular unique molecular identifier (UMI) counts. The “FindVariableGenes” function was applied to calculate highly variable genes by setting the mean expression in a range of 0.125 and 5 and the quantile normalized variance > 0.5. Then, based on principal component analysis (PCA), 20 essential principal components (PCs) were selected for uniform manifold approximation and projection (UMAP) dimensional reduction. All cells were clustered using the “FindClusters” function, and unbiased clustering yielded 27 major clusters (the top 25 principal components with a resolution of 0.5), annotated into 8 main cell types by manual annotation (well-known cell markers).

For further analysis, violin and dot plots were established via the “Vlnplot” and “Dotplot” functions, respectively. For heatmaps, the “DoHeatmap” function was applied using specific genes of each cell cluster/type. Cell–cell crosstalk was estimated using CellChat. The differentiation in the TME was mapped out via Monocle 3 to generate substantial translational links between different cell types. CytoTRACE was utilized to establish the differentiation potential [33].

### Transmission electron microscopy (TEM)

The tissue and cell TEM was carried out per the manufacturer’s protocol. Specimens were dyed with 0.3% lead citrate and photographed via an electron microscope (Hitachi, Tokyo, Japan; 2500 × or 30,000 × Magnification).

### Hematoxylin and Eosin (HE) staining

HE staining was used for histopathological assessment of tissues. Fresh samples were fixed for 24 h in 10% formalin before being embedded in paraffin. Tissue slides were baked at 60 °C for 1 h, then deparaffinized with dimethylbenzene, hydrated with gradient ethanol, stained with HE, dehydrated with gradient ethanol, and mounted with neutral balsam. After sealing sections with neutral balsam, photos were taken and analyzed using an upright epifluorescent microscope (Nikon, Eclipse Ni-E, Tokyo, Japan).

### Immunohistochemical staining (IHC)

Tissues were embedded in paraffin, sliced, and mounted onto slides. IHC was conducted according to a standardized protocol [34]. IHC score, also known as the H-score, was calculated as previously described [35]. Two pathologists independently performed the pathological diagnosis.

### Multicolor immunofluorescence and co-expression analysis

The multicolor immunofluorescence assessment was based on the tyramide signal amplification (TSA) system. Briefly, the sliced tissue specimens were dewaxed, rehydrated, treated for heating-induced epitope retrieval (HIER) with hydrogen peroxide, blocked using 3% BSA to inhibit nonspecific interaction, and labeled with primary antibodies and horseradish peroxidase-conjugated anti-rabbit secondary antibodies and fluorescent tyramide consecutively. Then, sections were treated for HIER, BSA blocking, and antibody staining. Next, nuclei were dyed with 4,6-Diamidino-2-phenylindole (DAPI) and imaged under a fluorescence microscope (Nikon, DS-QilMC, Tokyo, Japan).

### Cancer-associated fibroblasts (CAFs) and normal fibroblasts (NFs) isolation

Primary human colorectal CAFs and NFs were separated as previously described [36]. NFs were separated from a normal colon approximately 20 cm from the tumor, and CAFs were extracted from a moderately differentiated adenocarcinoma from a surgical specimen of the same patient. The two tissues were sliced into about 1 mm^3^ pieces and washed thrice with PBS and antibiotics (penicillin [100 U/ml], streptomycin [100 µg/ml], and gentamycin [50 µg/ml]). Next, 0.1% type III collagenase with deoxyribonuclease (DNase) I (30 U/ml) was utilized for tissue digestion at 37 °C for 0.5 h in a water bath, followed by filtering via a 70‐μm filter to remove undigested debris, and then centrifuged briefly. CAFs and NFs were cultivated in supplemented DMEM (10% fetal bovine serum [FBS], 100U/ml penicillin, 100 µg/mL streptomycin) at 37 °C with 5% CO_2_. The characteristics of tissue-derived fibroblasts were verified by their morphology and the expression of fibroblast markers and cell morphology. The precise details are given in the Supplemental Material Fig. S1.

### Cell culture

The human CRC RKO (Cat: TCHu116) and SW480 (Cat: SCSP-5033) cell lines were acquired from a 113-cell repository of the China Academy of Sciences (Shanghai, China) and propagated in DMEM augmented with 10% FBS. All cells were cultured in 5% CO_2_ at 37 °C. Moreover, the identity of the cell lines was confirmed and the cell’s mycoplasma contamination was successfully carried out using the short tandem repeat (STR) profiling method.

### Lentiviral vector and plasmid construction and transfection

All plasmids and lentiviruses were designed and constructed by GeneChem. Detailed information on the construction of various plasmids and the production of lentiviruses and plasmids is presented in Supplemental Tables S3–6 and S7–10. The transfection protocol was carried out per the manufacturer’s protocol. The accuracy of transduction, knockdown, and overexpression was assessed via western blotting (WB) (Figure S2A-B). The protocol with the highest transfection effectiveness was selected.

### Establishment of a co-culture unit

For tumor/CAF co-culture analysis, the passage number of primary CAFs of < 11 was utilized. A transwell technique was applied to propagate CRC cells and CAFs in a non-contact co-culture unit. The cultural media was refreshed once every 48 h. After 5 days, cells were harvested from the bottom compartment for subsequent analysis [37].

### Wound healing assay

The RKO and SW480 cells were propagated for the wound-healing assays to assess migration capabilities. Cells (4 × 10^5^/well) in 6-well plates were propagated for 24 h in a serum-free medium. Then the medium was discarded, and wounds were created in the cell layer using a 200-μL pipette tip. The wound healing rate was assessed at 0, 12, and 24 h under a phase-contrast microscope (Olympus CKX 41, Olympus, Hachioji, Japan) at 200 × magnification.

### Transwell assay

A transwell assay was carried out to assess the invasion ability of the cells as previously described [38]. Cells (5 × 10^4^) were propagated in DMEM (200 μL) in the upper chambers of transwell 24-well plates with a pore size of 8 µm. A chemoattractant was inoculated in a medium (500 μL) augmented with 10% FBS in the lower chambers. Matrigel was laminated on the transwell chambers. After 24 h of co-culturing, the upper transwell chamber was rinsed using 1% PBS, and the chambers were preserved for 15 min with 4% paraformaldehyde before staining with 0.1% crystal violet. Cells that invaded the lower chambers were imaged under a phase-contrast microscope (Olympus, CKX 41, Hachioji, Japan), and cells were quantified using ImageJ.

### WB assay

The WB assay was performed as previously described [27]. The radioimmunoprecipitation (RIPA) buffer was utilized for cell lysis to acquire proteins, which were quantified via Bradford assay. Approximately 20 µg of each sample was isolated by sodium dodecyl-sulfate polyacrylamide gel electrophoresis (SDS-PAGE) (8 or 10%), transplanted on the polyvinylidene fluoride (PVDF) membrane, blocked in BSA (5%), probed with appropriate primary antibodies at 4 °C overnight, rinsed thrice using Tris-buffered saline + Tween-20 (0.05%), and then labeled with the corresponding secondary antibodies. β-actin/GAPDH proteins were utilized as reference.

### Xenograft tumor model

The in-vivo experiments were authorized by the Animal Ethics Committee of Jiangsu Province Hospital of Chinese Medicine (2022DW-10–01). This research followed the Animal Research: Reporting of In Vivo Experiments (ARRIVE) guidelines (https://arriveguidelines.org). Nude male BALB/c mice (4 weeks old, 18–22 g) were bought from Beijing Weitong Lihua Experimental Animal Technology Co., Ltd. (Certificate No. SYXK2019-0010). The mice were subcutaneously administered in the right armpit with CAFs (2 × 10^6^) and SW480 (2 × 10^6^). CAFs were stably transfected with sh-*LMOD1*, oe-*LMOD1*, small interfering RNA targeting fibroblast growth factor 1 (si-FGF1), and normal control (NC) cells. To explore the tumor-promoting potential of the A-kinase anchor protein 1 (AKAP12)/LMOD1 axis in vivo, SW480 (4 × 10^6^) was inserted in the right flank of nude mice. SW480 cells were stably transfected with sh-AKAP12, oe-AKAP12, oe-LMOD1, and NC. Six mice were used for each experimental group. After 7 days, the existence of tumors was identified. The largest and smallest diameters of the tumor were assessed twice weekly, and on day 28, mice were sacrificed using CO_2_ according to the American Veterinary Medical Association (AVMA) Guidelines for Humane Animal Euthanasia [39]. The sera were collected, and tumors were sampled for volume analysis using the following formula: V = 1/2ab^2^ (“a” and “b” represent tumor length and width in mm). Growth curves were generated.

### Statistical analysis

The correlation between variables was assessed using the Pearson and Spearman correlation coefficients. The statistical significance of non-normally and normally distributed variables was assessed using the Mann–Whitney U test (also called the Wilcoxon rank sum test) and t-test, respectively. Kruskal–Wallis and one-way ANOVA tests were applied for inter-group comparison. Furthermore, the contingency table was elucidated using a two-sided Fisher’s exact test. The Kaplan–Meier method was applied to generate survival curves for subgroups, and statistically significant differences were elucidated using the log-rank (Mantel-Cox) test. The hazard ratio (HR) was identified using the univariate Cox proportional hazard regression model. All statistical analyses were carried out using R, with a P < 0.05 (two-tailed) depicting significant differences.

## Results

### Single-cell atlas of epithelial tissue, intestinal adenoma, and CRC

To characterize colorectal precancerous and malignant lesions, 9 biopsies were performed, comprising 3 pairs of normal intestinal epithelial biopsies (3 cases), adenoma biopsies (3), and CRC biopsies, respectively, from the same patient (Figure S3A, Table S11). All CRC samples were pathologically diagnosed as moderately differentiated (G2), and no other primary tumors were found. For each sampling, individual cells were selected without prior cell filtering, and sequencing data were generated using a 10X chromium platform. After removing low-quality cells, 20,262 high-quality cells were retained for subsequent analysis. To assess different cell types based on gene transcription profiles, downscaling and unsupervised cell clustering were carried out using the Seurat package after removing the batch effect among multiple samples. As shown in t-distributed stochastic neighbor embedding (t-SNE) and UMAP, 26 major cell clusters were finally identified in all samples (Figure S3B), which were then defined as single-cell transcriptome profiles of normal-precancerous lesions and CRC. These clusters were categorized by marker genes into eight known cell lines: T cell (marked by *CD3D*), plasma B cell (marked by *MS4A1*), monocyte (marked by *S100A9*), epithelia cell (EC, marked by *EpCAM*), follicular B cell (marked by *MZB1*), Macrophage (marked by *CD14*, *CD163*, and *CD68*), mast cell (marked by *KIT*), and fibroblast (marked by *DCN*) (Figure S3C-D). In addition to the typical cell type indices, other genes that marked each cell type were identified (Fig. S3E). The distribution of different samples and different tissue types on UMAP, respectively, is depicted in Fig. S3F and G. Each cell lineage proportion considerably differed among different samples (Fig. S3H), suggesting strong heterogeneity.

### Genetic characterization and transcriptional variation of CC

The stability of the tissue microenvironment depends on the mutual communication of different cells, and various mechanisms have evolved for this purpose, of which the most direct and efficient is through channels that directly connect the cytoplasm of neighboring cells [40, 41]. TJs and GJs are the two most classical modes of communication, and their dysfunction is associated with the development of various diseases, especially tumors [42, 43]. Our previous study showed that gap junction protein alpha 4 (Cx43, also named GJA4) is lowly expressed in normal intestinal mesenchymal tissues but highly expressed in CRC mesenchymal tissues and may impair the survival of CRC patients through CAF-related pathways [44]. Building on this finding, we further incorporated TJs into our analysis to explore the significance of CC in CRC from a more comprehensive perspective.

**Fig. 1:**
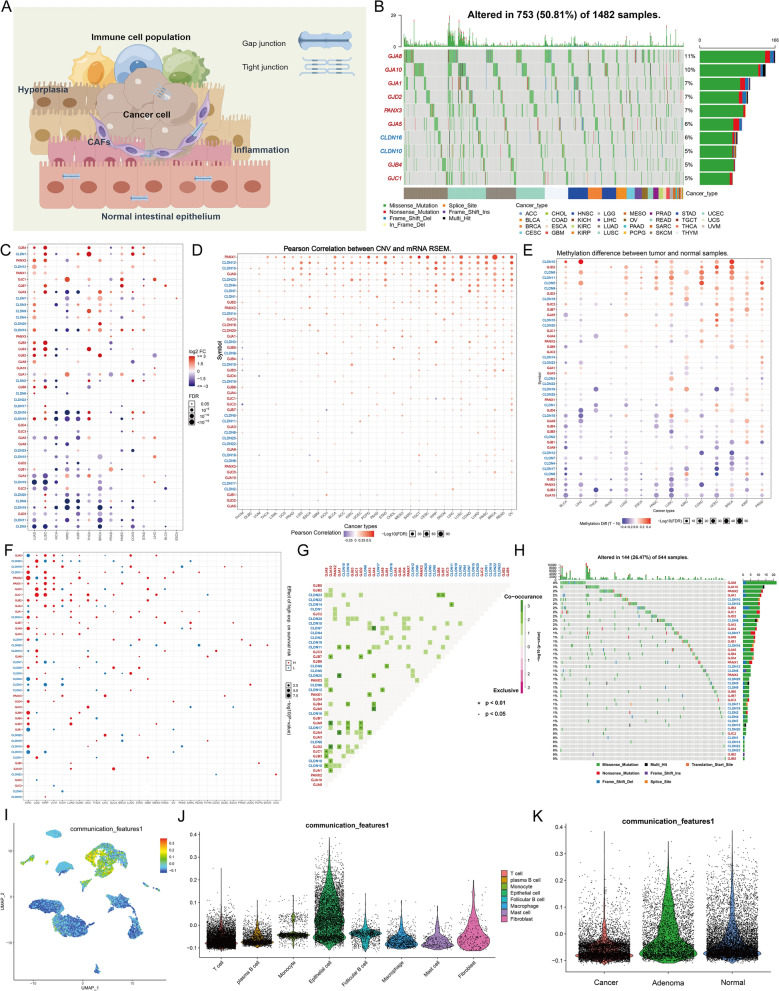
Comparison of expression levels of cell communication (CC) molecules. **A** Patterns mapped on the BioRender website to reveal the regulatory mechanisms of CC in tumors and their functions in the immune microenvironment of tumors. **B** The waterfall diagram illustrates the most frequent 47 CC molecule’s somatic mutations in The Cancer Genome Atlas (TCGA) pan-cancer data. 50.81% represents the proportion of 753 samples with at least 1 mutation of the top 10 genes among 1482 samples with at least one mutation of 47 CC genes. The percentage value on the right side of each line in the image indicates the number of samples with the specific gene mutation divided by 1,482 samples which had at least one mutation among the 47 CC genes. We label different types of CC molecules in red [gap junctions (GJs)] and blue [tight junctions (TJs)], respectively. **C** The dot’s color = degree of fold change. Red = high and blue = low expression in cancer tissue. Fold change = mean (tumor)/mean (normal), p-values were used. Field realistic doses (FDR) was utilized for adjusting the t-test and p-value. The size of the bubble indicates FDR; the larger the bubble, the lower the FDR. Genes with > twofold change and significance (FDR > 0.05) were used to plot graphs. If no significant genes are present in a cancer type, that cancer type was not included in the final figure. **D** Bubble plots display the correlation between the Copy number variant (CNV) (D) as well as DNA methylation (**E**) and the expression of the mRNA levels. A positive correlation is reflected in red, while a negative correlation is indicated by blue. Darker colors indicate a higher correlation index. The FDR is indicated by the bubble size. **F** Bubble plots showing the results of a log-rank test of the survival of 47 CC molecules in the TCGA-CRC cohort. Red represents detrimental to survival and blue denotes favorable to survival. The FDR is represented by the bubble size. **G** The mutation profiles of 44 CC molecules in 544 CRC patients in the TCGA-CRC cohort; co-mutations are shown by the green, mutex-mutations are indicated by the red, and asterisks indicate P values (*P < 0.05,^**.**^P < 0.01). **H** Mutation frequency of 47 CC molecules in 544 CRC patients in the TCGA-CRC cohort. The small graph above is the Tumor Mutational Burden (TMB), and the numbers on the right indicate the mutation frequency of each gene and provide the proportion of each variant. **I**, **J** Uniform Manifold Approximation and Projection (UMAP) (**I**) and violin (**J**) plot indicates the CC feature level (generated by the “AddModuleScore” function) across different cell types in our single cell RNA data. **K** Violin plot showing the CC feature level across different tissue types

Information on the 47 CC molecules was acquired from the HUGO gene Nomenclature Committee portal, including 24 GJ proteins (connexins and pannexins) and 23 TJ proteins (CLDNs). The localization and regulatory mechanisms of CC in the TME are presented in Fig. 1A. To detect genetic variation in cancer cell communication molecules, 1,482 samples were selected with at least one mutation per 47 CC molecules in the TCGA pan-cancer dataset. The oncoplot revealed the most frequent somatic mutations linked with 10 CC molecules in pan-cancer tissues. Of the 1482 samples, mutations were found in 753 cases, with a mutation frequency of 50.84%. It was revealed that *GJA8* exhibited the highest mutation frequency (11%), followed by *GJA10* (10%) and *GJA1* (7%). Moreover, missense mutation was the most frequent nutation, and ovarian cancer (TCGA-OV) was the most frequent mutated cancer (Fig. 1B). Considering that only 14 types of cancer indicated > 10 paired tumors and normal samples, transcriptome differential expression was compared between these cancers. Further analysis of messenger RNA (mRNA) levels of the 47 CC molecules revealed that the expression of various genes was reduced in several tumor tissues, including *CLDN5*, *CLDN11*, *GJD3*, *CLDN19*, and *CLDN20* (Fig. 1C). These findings indicated that genetic variation is one of the essential factors that influence CC expression. Most cancers revealed a positive relationship between copy number variant (CNV) and mRNA expression levels, especially *PANX1* (Fig. 1D). Intriguingly, mRNA expression levels were negatively associated with DNA methylation in a subset of cancer types, with an opposite trend in another subset (Fig. 1E). In addition, the prognostic significance of different CC molecule expressions varied across different types of tumors (Fig. 1F). Meanwhile, transcriptional patterns of CC molecules were markedly heterogeneous in normal and various cancer samples, suggesting a correlation between aberrant expression and transcriptional variants.

Due to the previous research base, the present study focused on CRC [44]. The TCGA database revealed substantial co-mutations between *CLDN23* and *GJB6*, *CLN23* and *GJB7*, *CLDN14* and *CLDN11*, *CLDN7* and *CLDN17*, etc. (P < 0.01, Fig. 1G). Of the 544 samples, 144 displayed CC molecular mutations, with a mutation frequency (26.47%). Among the 47 genes, *GJA8* had the highest mutation frequency (4%) (Fig. 1H), which was the missense mutation. The correlation among CC molecules in TCGA-CRC is shown in Figure S4. To characterize the localization of CC molecules at the single-cell level, the expression of CC molecules was scored using the “AddModuleScore” function of the “Seurat” package in our scRNA data. The results showed that the CC molecules were up-regulated in ECs (Fig. 1I, J) and significantly down-regulated in cancerous tissues (Fig. 1K), compared with normal intestinal epithelium and intestinal adenomas. These results suggested that an imbalance of CC molecules may lead to CRC.

### TJs are involved in the malignant transformation of intestinal epithelial cells (IECs)

**Fig. 2:**
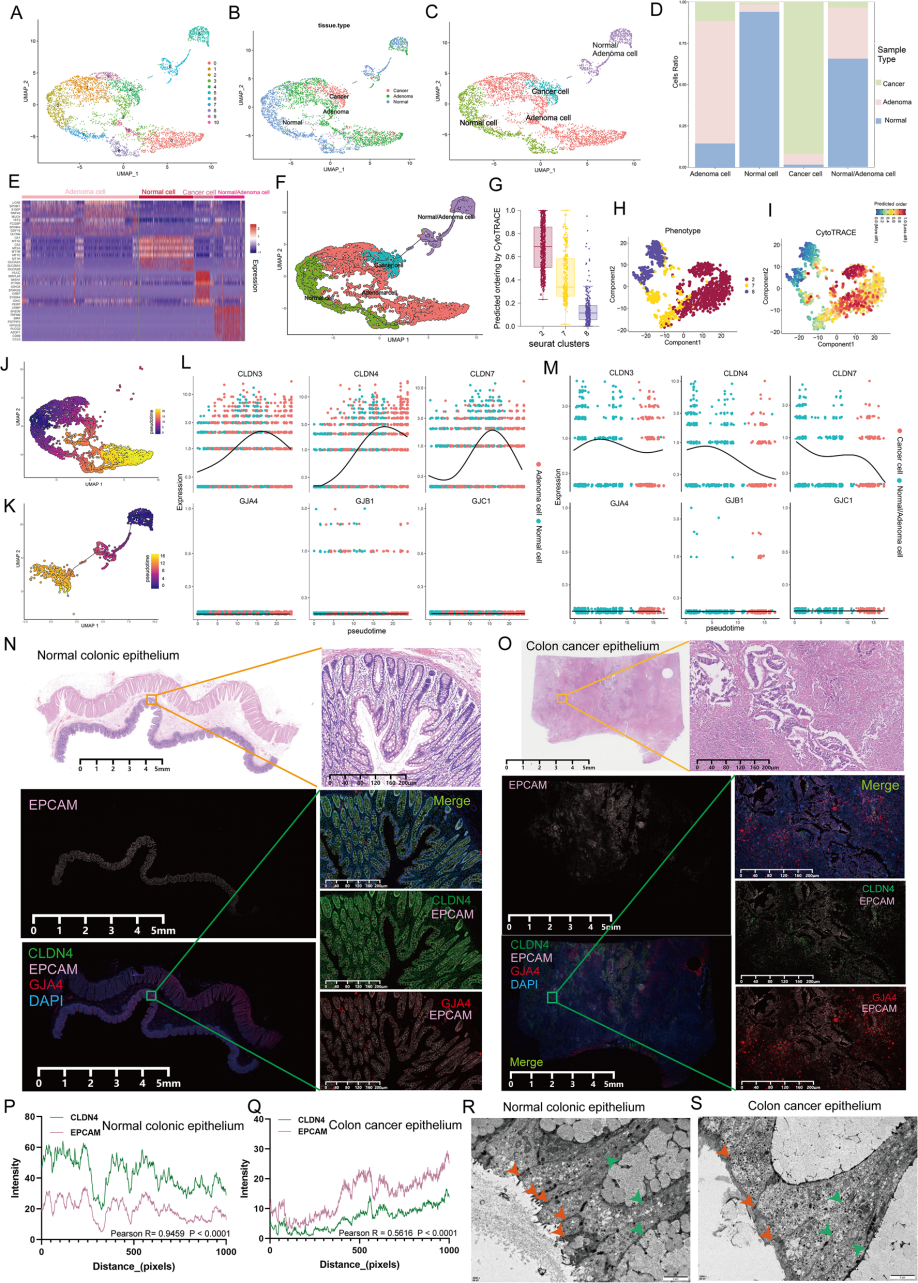
Heterogeneous landscape of CC molecules across different lesions in epithelial cells. **A**–**C** UMAP plot of all epithelial cells, color-coded for eleven seurat clusters (**A**), three tissue types (**B**), and four cell types (**C**). **D** The fraction of three tissue types in four cell types. **E** Heatmap showing differentially expressed genes among the four cell types (fold change > 1.5, FDR < 0.01). **F** The trajectories of all epithelial cells constructed by Monocle 3. Each point corresponds to a single cell and is colour coded by pseudotime. **G**–**I** Box (**G**) and t-distributed stochastic neighbor embedding (t-SNE) (**H**, **I**) plot demonstrate the degree of differentiation of cluster 2, 7, and 8 (three normal epithelial cell clusters) assessed by CytoTRACE. **J**–**K** Monocle 3 demonstrates two trajectories of cellular differentiation present in epithelial cells, including from normal cells to adenoma cells (**J**) and from normal/adenoma cells to cancer cells (**K**). **L**, **M** Two-dimensional plots showing the dynamic expression of representative CC molecules during the epithelial cell transitions during the pseudotime. **N** Multiplex immunofluorescence (mIF) staining images of CLDN4 (green), EPCAM (pink), and GJA4 (red) in a resected normal colon specimen (blue, DAPI), and CLDN4 (for TJs) is upregulated in epithelial cells (marked by EPCAM). Scale bars are labeled on the graph. **O** mIF staining images of CLDN4 (green), EPCAM (pink), and GJA4 (red) in a resected colon cancer specimen (blue, DAPI), And CLDN4 (for TJs) is downregulated in epithelial cells (marked by EPCAM). Scale bars are labeled on the graph. **P**, **Q** Co-localization was determined using the Pearson correlation coefficient in normal colon specimens (P, R = 0.9459, P < 0.0001) and colon cancer specimens (Q, R = 0.5616, P < 0.0001), respectively. The co-localization relationship between CLDN4 and EPCAM was weaker in tumor tissue compared to that of normal tissue. The X-axis represents each pixel point on the image, and the Y-axis represents the gray value corresponding to each pixel point. **R**, **S** The ultrastructure of junctions was examined using transmission electron microscope (EM) in in normal colon specimen and colon cancer specimen. Orange arrows indicate TJs and green arrows denote GJs

The dynamic changes in molecular signaling during EC malignant transformation (a protagonist in TME) have attracted considerable attention [45, 46]. Our scRNA data showed that CC molecules had the highest expression levels in ECs compared with other cells (Fig. 1I, J). After subclustering all ECs, 11 distinct groups were identified (Fig. 2A). These cell fractions were assigned to four major cell types, including normal, adenoma, cancer, and normal/adenoma cells, based on the type of tissue origin of each cell (Fig. 2B–D). Specific genetic markers for each cell type are displayed in Fig. 2E, further reflecting a high degree of heterogeneity of ECs. Then, cell trajectory analysis was carried out using the Monocle toolkit to further elucidate possible evolutionary routes between cell types. The pseudotime trajectory axis based on transcriptional profiles revealed two distinct trajectories of transdifferentiation (Fig. 2F). Using CytoTRACE, Cluster 2 (high CytoTRACE score) was identified as the normal-lesion transdifferentiation initiation point (Clusters 2, 7, and 8 were predominantly derived from normal tissues, Fig. S5A and Fig. 2G–I). Moreover, normal IECs were further classified into different functional subpopulations based on specific gene markers (Figure S5B). It was found that the two routes had distinct characteristics; specifically, a fraction of normal IECs (Cluster 2, Cluster 7, and Cluster 8) transdifferentiated into adenoma cells, whereas another fraction (mixed with adenoma cells) eventually transdifferentiated into cancer cells (Fig. 2J, K). To characterize the function of CC during transdifferentiation, *CLDN3*, *CLDN4*, and *CLDN7* were selected to represent TJs, and *GJA4*, *GJB1*, and *GJC1* were selected to represent GJs, respectively. Pseudotemporal expression dynamics of the representative genes showed that TJ levels increased and then decreased during normal-adenoma transdifferentiation and decreased directly during normal-cancer transdifferentiation. However, no change in both transdifferentiation routes was observed in GJs, which were always maintained at low levels (Figs. 2L, M and S6, S7). Further, multiplex immunofluorescence (mIF) staining of independent normal and cancer resection specimens confirmed that epithelial TJ levels were down-regulated in CRC tissues (as evidenced by an attenuated pattern of co-expression of EC-specific marker proteins epithelial cellular adhesion molecule [EpCAM] and CLDN4), whereas GJ levels did not differ significantly between normal intestinal epithelium and CRC tissues (Fig. 2N–Q). Loss of CLDN expression usually accompanies TJ destruction during tumor progression and leads to the acquisition of a malignant phenotype in cancer cells [47, 48]. Thus, the ultrastructure of CRC cells was observed under TEM. Compared with normal controls, CRC cells exhibited reduced TJ-mediated barrier formation and loose junction structures (Fig. 2R–S). These findings suggest that TJs are involved in the transdifferentiation of IECs to adenoma and carcinoma cells.

### GJs are involved in the malignant transformation of fibroblasts

**Fig. 3:**
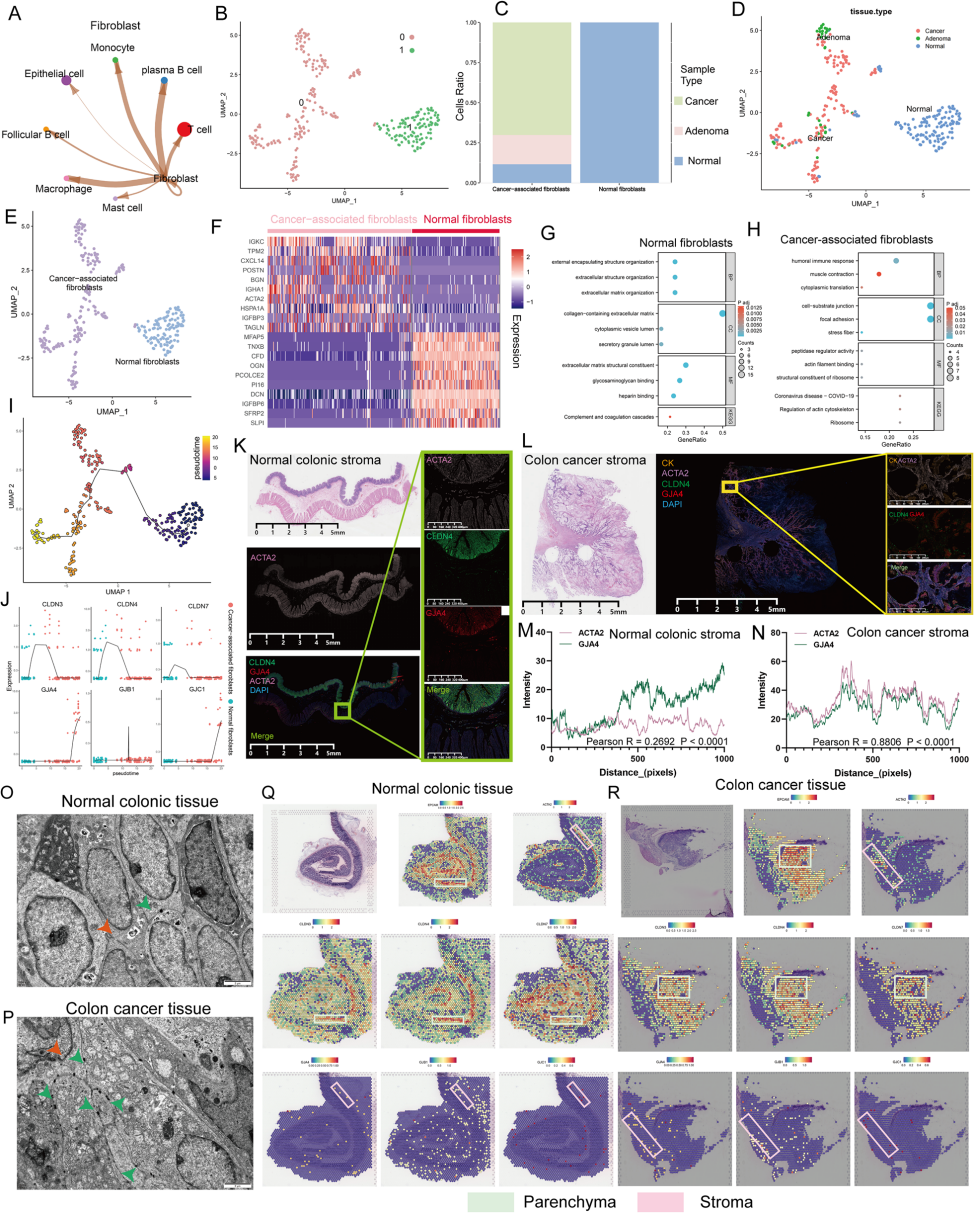
The heterogeneous landscape of CC molecules across different lesions in fibroblasts. **A** Circle plot showing the possible ligand-receptor pairs between fibroblasts and other type cells (predicted by CellChat). **B** The UMAP plot of all fibroblasts, color-coded for two seurat clusters. **C**, **D** The fraction of three tissue types in two cell types showed by histogram (**C**) and UMAP plot (**D**). **E** All fibroblasts were defined as Normal fibroblasts (NFs) and Cancer-associated fibroblasts (CAFs), respectively, according to tissue origin. **F** Heatmap showing differentially expressed genes between the two cell types (fold change > 1.5, FDR < 0.01). **G**, **H** The bubble plots indicate the up-regulated gene set in NFs (**G**) and CAFs (**H**), differently. **I** Trajectory of fibroblasts constructed by Monocle 3. Each point corresponds to a single cell and is colour-coded by pseudotime. **J** Two-dimensional plots showing the dynamic expression of representative CC molecules during the fibroblast transitions along the pseudotime. **K** mIF staining images of ACTA2 (pink), CLDN4 (green), and GJA4 (red) in a resected normal colon specimen (blue, DAPI), and GJA4 (for GJs) is lowly expressed in normal mesenchymal tissues (marked by ACTA2). Scale bars are labeled on the graph. **L** mIF staining images of CK (orange), ACTA2 (pink), CLDN4 (green), and GJA4 (red) in a resected colon cancer specimen (blue, DAPI), and GJA4 is highly expressed in cancer mesenchymal tissues (marked by ACTA2 and CK). Scale bars are labeled on the graph. **M**, **N** Co-localization was evaluates based on the Pearson correlation coefficient in normal colon specimens (M, R = 0.2692, P < 0.0001) and colon cancer specimen (N, R = 0.8806, P < 0.0001), respectively. The co-localization relationship between GJA4 and ACTA2 was stronger in tumor tissue compared to normal tissues. The X-axis represents each pixel point on the image, and the Y-axis represents the gray value corresponding to each pixel point. **O**, **P** The ultrastructure of junctions in normal colon specimens and colon cancer specimen was examined using a transmission electron microscope (EM). Orange arrows represent TJs, and green arrows represent GJs. **Q**, **R** Spatial transcription sections indicate the spatial expression of *EPCAM*, *ACTA2*, TJs markers (*CLDN3/4/7*), and GJs markers (*GJA4*, *GJB1*, and *GJC1*) in normal colonic tissue (**Q**) and colon cancer tissue (**R**). The dot color indicates the expression level of the markers. Green boxes for the parenchyma and pink boxes for the mesenchyme

CAFs are important tumor stroma components [49, 50]. The signaling crosstalk that develops between CAFs and other cells is necessary to maintain the TME [51, 52] (Fig. 3A, estimated using CellChat in our scRNA data). Here we further established the expression atlas of CC features in fibroblasts. Cluster analysis of all fibroblasts was performed, and two significant cell subpopulations (Cluster 0 and Cluster 1) were identified (Fig. 3B). Based on the proportion of single-cell tissue sources in the two clusters (Fig. 3C, D), CAFs were mainly represented by C0, indicating high expression of *BGN*, *POSTN*, and *ACTA2*, and NFs were mainly represented by C1, indicating high expression of *OGN*, *POSTN*, and *MFAP5* (Fig. 3E, F). Next, gene set enrichment analysis (GSEA) was performed to decipher the differences in molecular characteristics between CAFs and NFs. Compared with NFs, CAFs were associated with signaling pathways such as cell-substrate junction, focal adhesion, actin filament binding, and regulation of the actin cytoskeleton (Fig. 3G, H). Notably, CAFs were mostly derived from NFs, which were recruited to the tumor region and reprogrammed into the former by cancer cells secreting cytokines. To further investigate this ongoing process, a trajectory analysis of CAFs and NFs was conducted (Fig. 3I). Contrary to the phenomenon observed in ECs, GJs but not TJs were involved in the transdifferentiation of NFs to CAFs. Specifically, GJ genes were all up-regulated during this differentiation, while the levels of TJ genes remained unchanged (Figs. 3J and S8). mIF staining of independent samples also confirmed that GJ protein expression was markedly increased in tumor mesenchymal tissues (high expression of actin alpha 2 [ACTA2] and low expression of cytokeratin) than in normal mesenchymal tissues, whereas no difference was found in TJs (Fig. 3K–N). Meanwhile, a higher density of GJ channels was observed in the ultrastructures of tumor stroma (Fig. 3O, P, Green arrow). To spatially confirm these co-localization and co-expression relationships, normal and cancerous were further studied. Spatial transcriptomics sections from GEO database were distinguished between epithelium and stroma by pathological sections and specific gene markers (EpCAM for epithelium and ACTA2 for stroma). Similarly, the co-localization of TJs and parenchyma was weakened and the co-localization of GJs and stroma was strengthened in CRC compared with normal intestinal tissue (Fig. 3Q, R). Collectively, these data imply that GJs may be important in the activation of CAFs.

### Unsupervised learning identified two different CC-level patterns

**Fig. 4:**
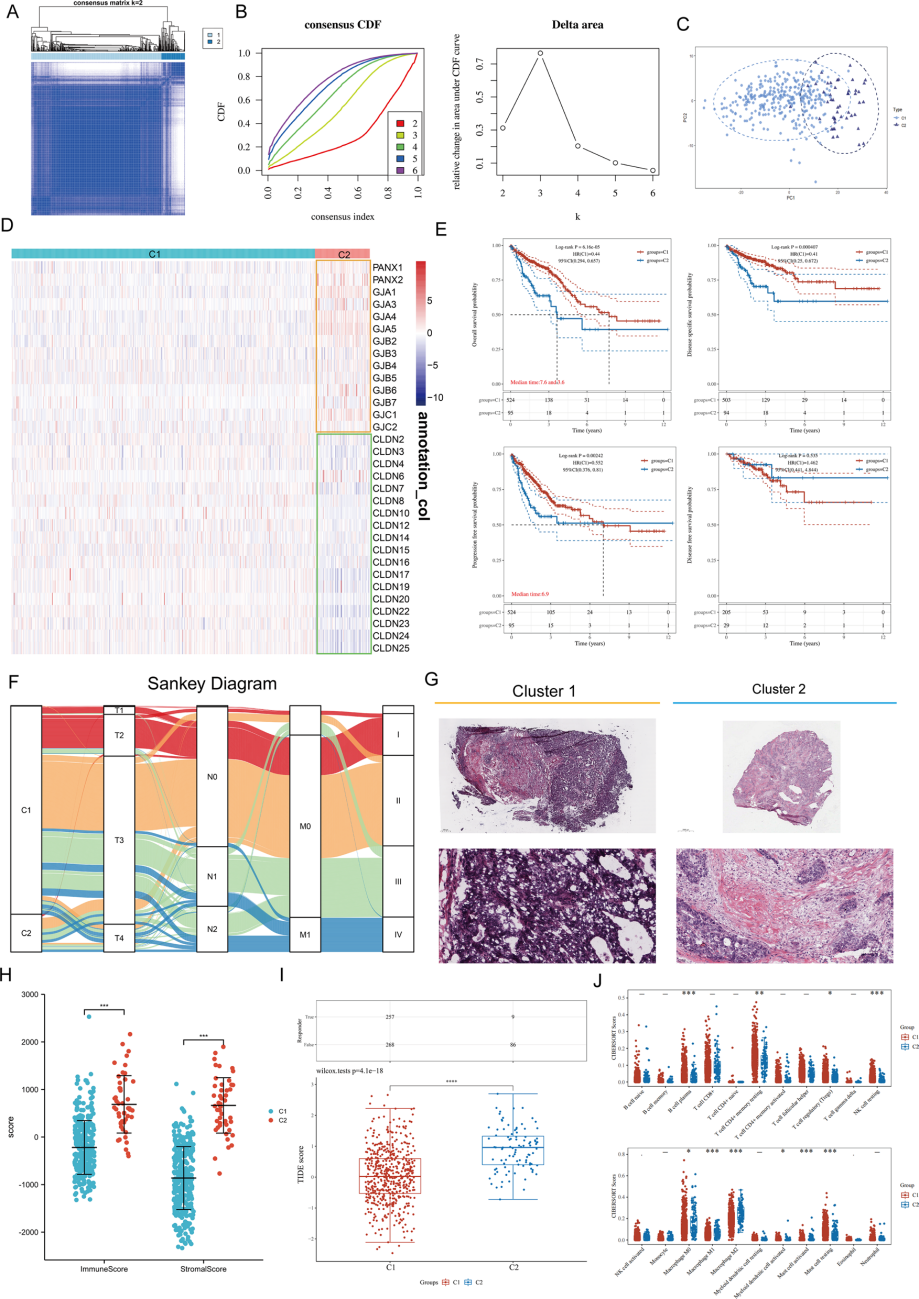
Unsupervised Machine Learning algorithms used to identify 2 molecular subtypes in TCGA-CRC. **A** Heat map showing the sample clustering at K = 2 (the optimal cluster number) in TCGA-CRC. **B** Left: The cumulative distribution function (CDF) curve in consensus cluster analysis. The consensus score’s CDF curves with various subtype numbers (k = 2, 3, 4, 5, and 6) are shown. Right: Relative change in area under the CDF curve for k = 2–6. **C** The TCGA-CRC samples were classified via Principal Component Analysis (PCA) based on the CC molecules expression profile. Different colors = C1 and C2 subtypes, respectively. Each point is a single sample. **D** The distribution of 47 CC molecules between two subtypes in TCGA-CRC. GJs molecules are upregulated in C2 and TJs molecules are upregulated in C1. **E** Survival analysis in terms of Overall Survival (OS), Disease-Specific Survival (DSS), Progression-Free Survival (PFS), and Disease-Free Survival (DFS) based on 2 subtypes (TCGA-CRC, Logrank test, n = 620). **F** The Sankey diagram completely showing the association between the subtypes and clinicopathological attributes. **G** Representative images of pathological Hematoxylin–eosin (HE) staining of 2 CC phenotypes (above, scale bars = 500 μm; below, scale bars = 50 μm). C2 contained a more abundant matrix component than C1. **H** Violin plots showing the immune score and stromal score of different CC patterns (Wilcoxon test). **I** The box plot indicating the Tumor Immune Dysfunction and Exclusion (TIDE) score of different CC patterns (Wilcoxon test). **J** Comparison of TME infiltrating cells between the two CC phenotypes (Wilcoxon test). ****P < 0.0001, ***P < 0.001, **P < 0.01, *P < 0.05

The above results suggested that a spatiotemporal heterogeneity of the CC molecules existed during malignant transformation. To comprehensively understand the integrated mechanism of the CC feature in CRC, unsupervised clustering was performed on 620 samples from TCGA-CRC. Two unique modification patterns were identified, named Cluster 1 (C1, 525 cases) and Cluster 2 (C2, 95 cases) (Fig. 4A, B). PCA confirmed that the two clusters were distinguishable by the 47 CC molecule expression levels (Fig. 4C). The thermogram showed that TJs had lower levels but GJs had higher levels in C2 than in C1 (Fig. 4D). Considering previous findings (Figs. 2 and 3), we speculated that the stroma of C2 might be in a more active state, while its epithelial component might have a weaker malignant feature. We analyzed survival prognosis differences between the two CC subtypes, and results showed that C1 had a distinct and significant survival advantage, whereas C2 had a poorer prognosis (OS, P = 0.047; DFS, P = 0.000407; DSS, P = 0.00242; PFS, P = 0.535; Fig. 4E). Next, the CC phenotype was compared with several commonly used clinical indicators. It was found that most C1 patients were in the advanced stages, whereas most C2 patients were in the early stages (Fig. 4F). Notably, the abundance of stromal components was lower in the tumor of C1 patients and higher in the tumor of C2 patients (TCGA pathology slides, Fig. 4G). The “ESTIMATE” method showed that C2 had high stromal and immune signals (Fig. 4H), both of which have been shown to correlate with poor outcomes [53, 54]. In addition, immunosuppressive stromal cells or abundant stroma hinder the success of immunotherapy. A computational method was applied to model the two main mechanisms of tumor immune evasion and tumor immune dysfunction and exclusion (TIDE) to predict the ICB response based on transcriptional profiles. The results showed that C2 had a significantly higher TIDE score than C1, suggesting that C2 patients may not benefit from immunotherapy (Fig. 4I, P < 0.0001). Immunological estimates indicated that M2 macrophages were highly enriched in C2 and that M2 macrophages are a major modulator of immune tolerance in cancer cells andconfer resistance to immunotherapy (Fig. 4J).

### Identification of LMOD1 as CC phenotype-associated factors

Differences in the gene expression between CC subtypes were assessed to identify potential regulators. A total of 157 DEGs were identified between C1 and C2 (Fig. S9A). Kyoto Encyclopedia of Genes and Genomes (KEGG) and Gene Ontology (GO) enrichment analyses revealed that the extracellular matrix (ECM) organization and collagen-comprising ECM, ECM structural constituent, and other collagen-matrigel matrix-related signals were significantly activated in C2 (Fig. S9B, Table S12). For hallmark enrichment, C2 was enriched for a large set of cancer-related genes, such as PI3K_AKT_MTOR_SIGNALING, MTORC1 SIGNALING, WNT_BETA_CATRNIN_SIGNALING, and NOTCH SIGNLING (Figure S9C). Afterward, a MEGENA network was established based on the DEGs (Figure S9D), and 8 modules and 157 module genes were obtained, with the largest module C1_5 encompassing 46 genes, followed by C1_2 with 33 genes and module C1_11 with 27 genes (Fig. S9E, Table S13). A Cox multivariable proportional hazards model constructed based on hub genes indicated that *COL1A2*, *LMOD1*, and *MYH11* were significant adverse factors for CRC (Fig. S9F–H, TCGA-CRC, PFI, P < 0.05). Considering the very limited number of studies on *LMOD1* in CRC, the current study focused on *LOMD1*, and Kaplan–Meier curves confirmed that *LMOD1* was associated with a worse DSS and PFI in CRC (Fig. S9I–K). Interestingly, there was a low expression of *LMOD1* in the CRC samples compared with the controls (Fig. S9L, P < 0.001). We further analyzed the difference in *LMOD1* gene expression among different clinical stages of CRC cases in the TCGA-CRC cohort and found a positive correlation (Figure S9M–P; T stage, P < 0.05; N stage, P < 0.01; M stage, P < 0.05; Pathologic stage, P < 0.05). Next, a network of *LMOD1*-related genes was generated in the GeneMANIA (http://www.genemania.org) and found a close link between *LMOD1* and mesenchymal-related genes, such as *ACTA2*, *ITGA1*, and *ACTG2* (Figure S9Q). Overall, these results indicated that *LMOD1* may be an important oncogene.

### LMOD1 resembles TJs in the epithelium and GJs in the stroma

**Fig. 5:**
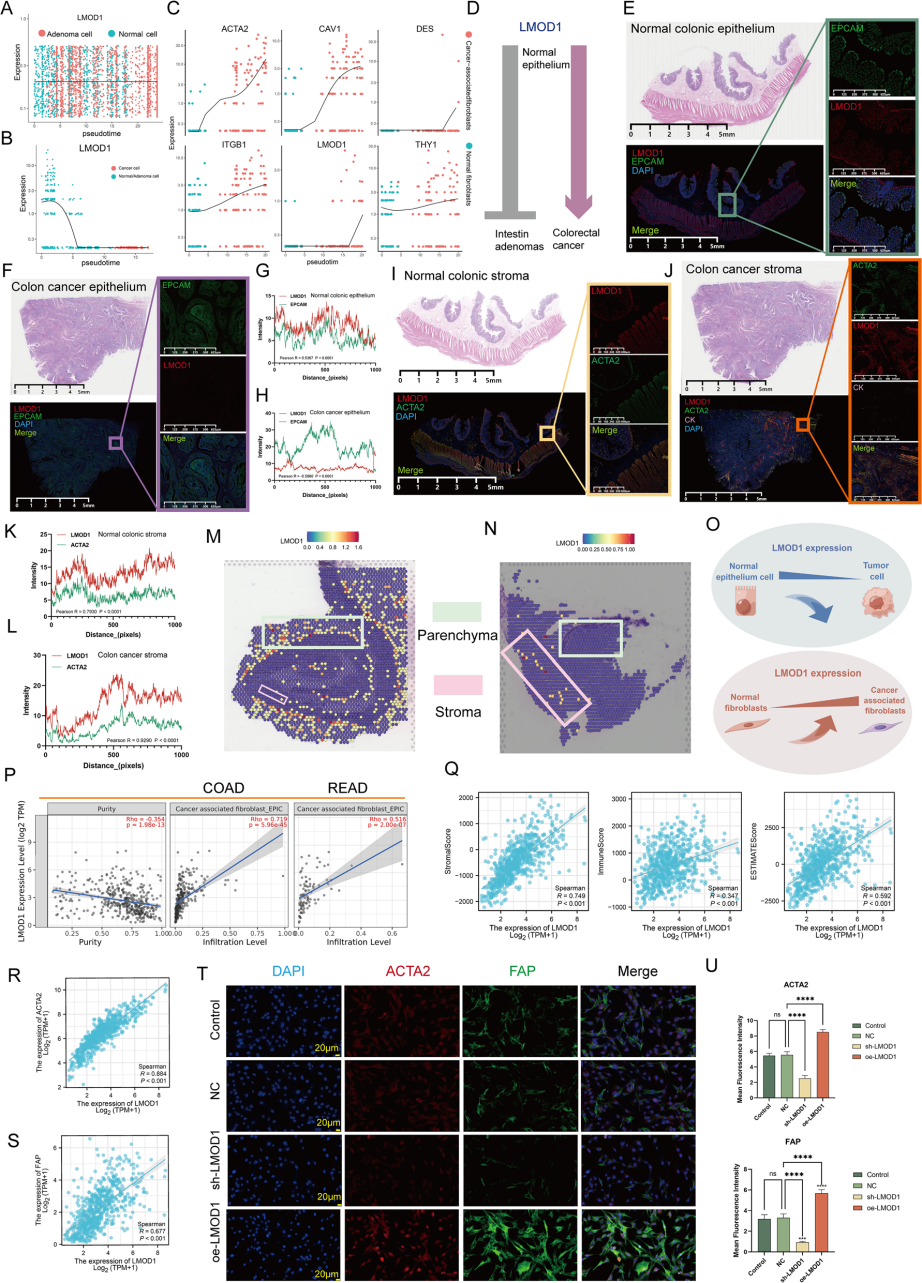
Transcriptional landscape heterogeneity of *LMOD1*. **A** Two-dimensional plots illustrating the invariabilities in *LMOD1* expression during the transitions (from normal cells to adenoma cells) along the pseudotime. **B** Two-dimensional plots showing the variations (decrease) in *LMOD1* expression during the transitions (from normal/adenoma cells to cancer cells) along the pseudotime. **C** Two-dimensional plots indicating the variations (increase) in *LMOD1* expression and fibroblasts activation markers during the transitions (from NFs to CAFs) along the pseudotime. **D** *LMOD1* is involved in transdifferentiation from normal epithelium to colorectal cancer but not from normal epithelium to intestine adenomas (grey⊥stands for no change; purple↓stands for decrease). **E**, **F** Double immunofluorescence (dIF) staining images of EPCAM (green) and LMOD1 (red) in a resected normal colon specimen (**E**) and a resected colon cancer specimen (**F**) (blue, DAPI). LMOD1 is upregulated in normal epithelial cells but downregulate in cancer cells. Scale bars are provided on the graph. **G**, **H** Co-localization was determined using the Pearson correlation coefficient in normal colon specimen (G, R = 0.5367, P < 0.0001) and colon cancer specimen (H, R = − 0.5860, P < 0.0001), respectively. The X-axis represents each pixel point on the image, and the Y-axis represents the gray value corresponding to each pixel point. The co-localization relationship between LMOD1 and EPCAM was weaker in tumor tissue compared to that of normal tissue, just like TJs. **I** dIF staining images of ACTA2 (green) and LMOD1 (red) in a resected normal colon specimen (blue = DAPI), and LMOD1 downregulated in NFs. Scale bars are labelled on the graph. **J** mIF staining images of ACTA2 (green), LMOD1 (red), and CK (pink) in a resected colon cancer specimen (blue = DAPI), and LMOD1 is upregulated in cancer mesenchymal tissues. Scale bars are provided on the graph. **K**, **L** Co-localization was determined using the Pearson correlation coefficient in normal colon specimen (K, R = 0.7930, P < 0.0001) and colon cancer specimen (L, R = 0.9290, P < 0.0001), respectively. The co-localization relationship between LMOD1 and ACTA2 was stronger in tumor tissue compared with that in normal tissue, simile to GJs. The X-axis represents each pixel point on the image, and the Y-axis represents the gray value corresponding to each pixel point. **M**, **N** Spatial transcription sections showing the spatial expression of *LMOD1* in normal colonic tissue (**M**) and colon cancer tissue (**N**). The dot color represents the expression level of the markers. Green boxes for the parenchyma and pink boxes for the mesenchyme. **O** LMOD1 exhibited similar behaviors as TJs during the malignant transformation of epithelial cells and to GJs in malignant transformation of fibroblasts. **P** Spearman correlation between *LMOD1* expression and the tumor purity (left) as well as infiltration level of fibroblasts in Colon adenocarcinoma (COAD) (middle) and Rectum adenocarcinoma (READ) (right) was analyzed on TIMER 2.0 (TCGA-CRC). **Q** Spearman association of *LMOD1* expression with stromal score (left), immune score (middle), and estimate score (right) was analyzed by “ESTIMATE” package (TCGA-CRC). **R**, **S** Spearman association of *LMOD1* with fibroblast activation markers *ACTA2* (**R**) and *FAP* (**S**) expression (TCGA-CRC). **T**, **U** Double staining technique by ACTA2 (green), and FAP (red) staining in the primary CAFs. Representative images of staining are shown. All assays were conducted thrice, independently (scale bars = 20 µm). ANOVA was applied. ****P < 0.0001

Furthermore, we explored the possible connection between *LMOD1* and CC features. Trajectory analysis (our scRNA data) showed that *LMOD1* was not involved in the transformation of normal epithelium into adenomas (Figs. 5A and S10A). Meanwhile, for the malignant transformation of ECs, the sequence of *LMOD1* expression changes was similar to that of TJs; for malignant transformation of fibroblasts, the sequence of *LMOD1* expression changes was in line with that of GJs and indicators of CAFs activation (Figs. 5B, C, S10B, and S11). This interesting pattern is summarized in Fig. 5D. mIF staining of independent samples confirmed that for the epithelium, *LMOD1* expression was lower in cancer tissues than in healthy tissues (Fig. 5E–H); the opposite result was observed for the stroma (Fig. 5I–L). In addition, we described the spatial distribution of *LMOD1* in the parenchyma and stroma of CRC tissues in the ST data (GEO database). As expected, *LMOD1* expression disappeared in the parenchymal component of the tumor (compared with normal parenchyma), whereas it was relatively increased in the tumor stromal component (Fig. 5M, N), as summarized in Fig. 5O. Subsequently, the Tumor Immune Estimation Resource (TIMER) tool and “ESTIMATE” package were employed for further quantification in TCGA-CRC. The results revealed a marked positive association between *LMOD1* expression and CAF infiltration level (colon adenocarcinoma [COAD], R = 0.719, P = 5.96e−45; rectum adenocarcinoma [READ], R = 0.516, P = 2.00e−07), and a significant negative correlation with tumor purity (R = − 0.354, P = 1.98e−13) (Fig. 5P). As depicted in Fig. 5Q, *LMOD*1 expression was significantly positively correlated with all the scores, especially the stromal score (R = 0.749, P < 0.001). Subsequently, we selected ACTA2 (Fig. 5R, TCGA-CRC, R = 0.884, P < 0.001) and fibroblast activation protein (FAP, Fig. 5S, TCGA-CRC, R = 0.677, P < 0.001) as markers of CAF activation. Human primary CAFs were successfully isolated, and it was confirmed in vitro that LMOD1 overexpression significantly promoted the expression of ACTA2 and FAP, whereas down-regulation of LMOD1 demonstrated the opposite result (Fig. 5T, U, P < 0.001).

### Fibroblast-expressing LMOD1 promotes cancer invasion through FGF1

**Fig. 6:**
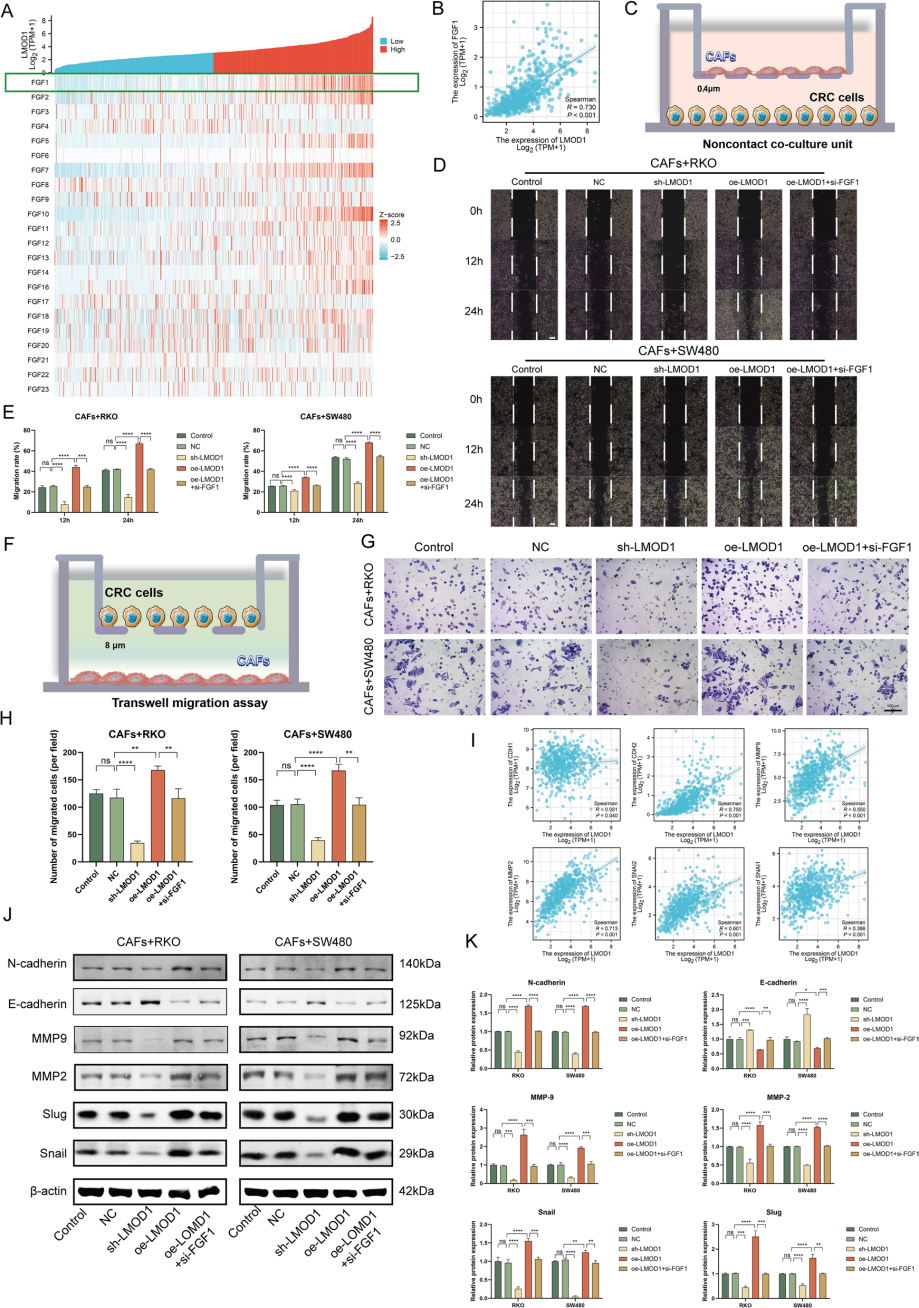
LMOD1/FGF1 in CAFs promotes CRC cell invasion and metastasis by regulating the EMT process. **A** Correlation analysis between *LMOD1* and fibroblast growth factors (FGFs) based on TCGA-CRC. **B** Pearsons’s correlation coefficient between *LMOD1* and *FGF1* expression based on TCGA data. **C** Non-contact co-culture unit of CAFs and CRC cells for cell migration (Wound healing assay). A 1:1 ratio of the cells was employed. **D**, **E** Cell migration (Wound healing assay) in RKO and SW480 cells with the intervention of CAFs over-/under-expressing LMOD1 in the presence or absence of si-FGF1. (Scale bars = 100 μm, magnification, × 200). **F** The cell invasion (Transwell assay) assay for non-contact co-culture unit of CAFs and CRC cells. A 1:1 ratio of the cells was employed. **G**, **H** Cell invasion (Transwell assay) for RKO and SW480 cells with the intervention of CAFs over-/under-expressing LMOD1 in the presence or absence of si-FGF1. (Scale bars = 100 μm, magnification, × 200). **I** Association of *LMOD1* with EMT-related genes based on TCGA-CRC. **J**, **K** The expression of EMT-associated proteins in RKO and SW480 cells as determined by western blotting (n = 3 replicates). Data are shown as mean ± SEM, ****P < 0.0001, ***P < 0.001, **P < 0.01, *P < 0.05. All assays were replicated thrice, independently

Since LMOD1 may play distinct roles in the epithelium and stroma, we investigated LMOD1 function in fibroblasts and ECs, respectively. A correlation assay indicated that *LMOD1* was positively correlated with most fibroblast growth factors (FGFs) (Fig. 6A, TCGA-CRC), especially *FGF1* (Fig. 6B, TCGA-CRC, R = 0.730, P < 0.001). FGF1 has been shown to play an important role in the expansion of CAFs by suppressing the transcription of tumor protein p53 (TP53) and triggering tumor fibrosis [55]. Importantly, CAFs form signaling crosstalk with cancer cells to support tumor growth. NFs co-evolve with cancer cells and transform into CAFs, and CAFs secrete cytokines to induce cancer cell survival [56]. Since we confirmed that LMOD1 promotes the activation of CAFs, we further investigated whether LMOD1 could stimulate cancer cell invasion through the above mechanism. A CAF-cancer cell co-culture model was established as shown in Fig. 6C. Under co-culture with oe-LMOD1-treated CAFs, RKO and SW480 cells exhibited more potent migration ability, and this effect was partially reversed by si-FGF1 (Fig. 6D, E, wound healing, P < 0.0001). Next, transwell experiments were performed in the model (Fig. 6F), which showed that LMOD1-treated CAFs affected the invasion ability of cancer cells (Fig. 6G, H, P < 0.01). Correlation analysis revealed that *LMOD1* may have a regulatory function on EMT-related markers (Fig. 6I: TCGA-CRC; *CDH2*, R = 0.750, P < 0.001; *CDH1*, R = 0.081, P = 0.040; *MMP9*, R = 0.550, P < 0.001; *MMP2*, R = 0.713, P < 0.001; *SNAI1*, R = 0.386, P < 0.001; *SNAI2*, R = 0.601, P < 0.001). The WB assay confirmed that LMOD1 up-regulation increased the levels of N-cadherin, matrix metalloproteinase 2 (MMP2), MMP9, Slug, and Snail in RKO and SW480 cells (co-cultured with CAFs in the pattern of Fig. 6C) but reduced the expression of E-cadherin; however, this effect was abolished by ectopic FGF1 expression (Fig. 6J, K). Together, these results suggest that LMOD1, localized on fibroblasts, is a proto-oncogene.

### AKAP12 regulates EC-expressing LMOD1 and inhibits *cancer* invasion

**Fig. 7:**
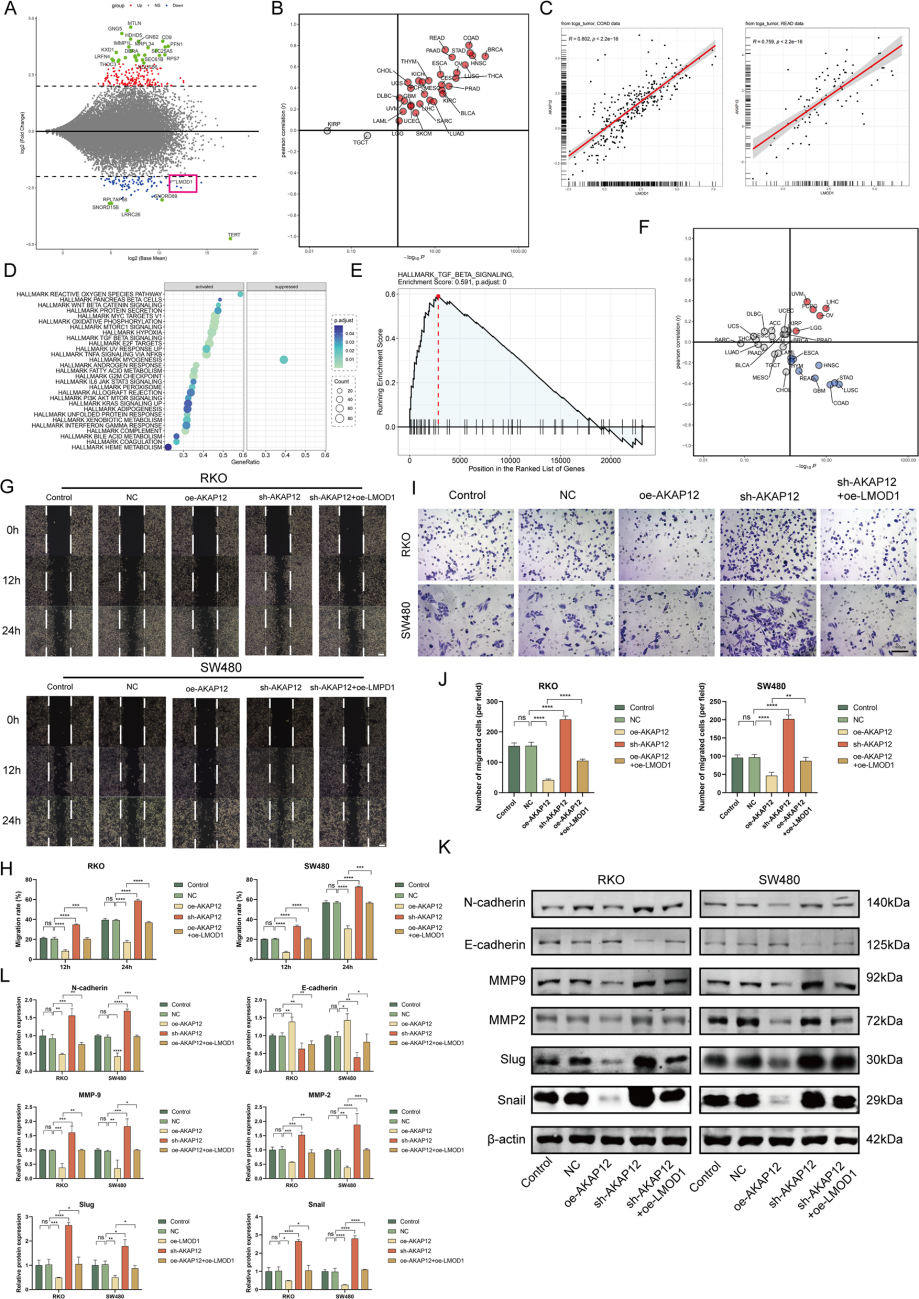
AKAP12/LMOD1 overexpression inhibits the malignant phenotype of CRC cells. **A** A volcano plot displaying the differentially expressed mRNAs in *AKAP12* knockdown cells [search in genetic perturbation similarity analysis database (GPSAdb), accession: GSE147739]. *LMOD1* was marked in a red box. **B** Association of *AKAP12* expression with *LMOD1* in TCGA pan-cancer. Grey = not statistically significant, Blue = negatively correlated, and Red = positively correlated. A spearman test was carried out. **C** Specific spearman correlation coefficients between *AKAP12* and *LMOD1* in TCGA-COAD (left) and TCGA-READ (right) are shown. **D** DEGs enrichment analysis after *AKAP12* knockdown in GSE147739. The larger the circle, the higher the number of genes, and the smaller the P-values, the darker the color and the more significant the enrichment. **E** Gene set enrichment analysis (GSEA) plots of TGF_BETA_BETA signals were analyzed in GPSAdb (accession: GSE147739). **F** Association of *AKAP12* expression with TGF_BETA_BETA signals level in TCGA pan-cancer. Blue = negatively correlated, Red = positively correlated, and Grey = not statistically significant. A spearman test was carried out. **G**, **H** The wound-healing assays for ROK and SW480 cells over-/under-expressing AKAP12 in the presence or absence of si-LMOD1 (Magnification, × 200, scale bars = 100 μm). **I**, **J** Transwell migration assays for ROK and SW480 cells over-/under-expressing AKAP12 in the presence or absence of si-LMOD1 (Magnification, × 200, scale bars = 100 μm). **K**, **L** The expression of proteins related to EMT in RKO and SW480 cells was determined by western blotting (n = 3 replicates). Data are presented as the mean ± SEM. ****P < 0.0001, ***P < 0.001, **P < 0.01, *P < 0.05. All assays were performed thrice, independently

Moreover, we investigated the function of LMOD1 in ECs. To predict the possible regulatory mechanisms of LMOD1 in epithelial tissues, we searched the GPSAdb database and found that interfering with *AKAP12* expression in SW480 cells significantly down-regulated *LMOD1* (Fig. 7A, gene perturbed data GSE147739, red box, P = 0.0007). In the TCGA pan-cancer database, *AKAP12* and *LMOD1* were positively correlated in most cancer types, suggesting the prevalence of a regulatory relationship between the two (Fig. 7B). Particularly, there was a high positive correlation between *LMOD1* and *AKAP12* in COAD (R = 0.820, P < 2.2e–16) and READ (R = 0.759, P < 2.2e-16) (Fig. 7C). The enrichment of the differential transcriptome generated by AKAP12 knockdown showed that AKAP12 was negatively associated with the activation of multiple cancer-related signaling pathways (Fig. 7D, E). Additionally, the TCGA pan-cancer data revealed that *AKAP12* expression and transforming growth factor-β (TGF-β) signaling were negatively correlated in CRC (Fig. 7F, COAD and READ). Considering that TGF-β signaling is a classical EMT inducer, we believe that these data strengthen the evidence for *AKAP12* and *LMOD1* as tumor suppressor genes.

Whether AKAP12/LMOD1 signaling could inhibit cancer cell metastasis was then investigated in vitro. Wound healing (Fig. 7G, H) and transwell (Fig. 7I, J) assays demonstrated that overexpression of *AKAP12* significantly inhibited cell migration and invasion, which was abrogated by ectopic LMOD1 expression. In addition, the WB assay showed that *AKAP12* down-regulation reduced E-cadherin expression in CRC cells but enhanced the expression of N-cadherin, MMP2, MMP9, Slug, and Snail; however, this effect was significantly attenuated after the rescued of LMOD1 expression (Fig. 7K, L).

### LMOD1 is biphasic in vivo and is associated with immunity to CRC

**Fig. 8:**
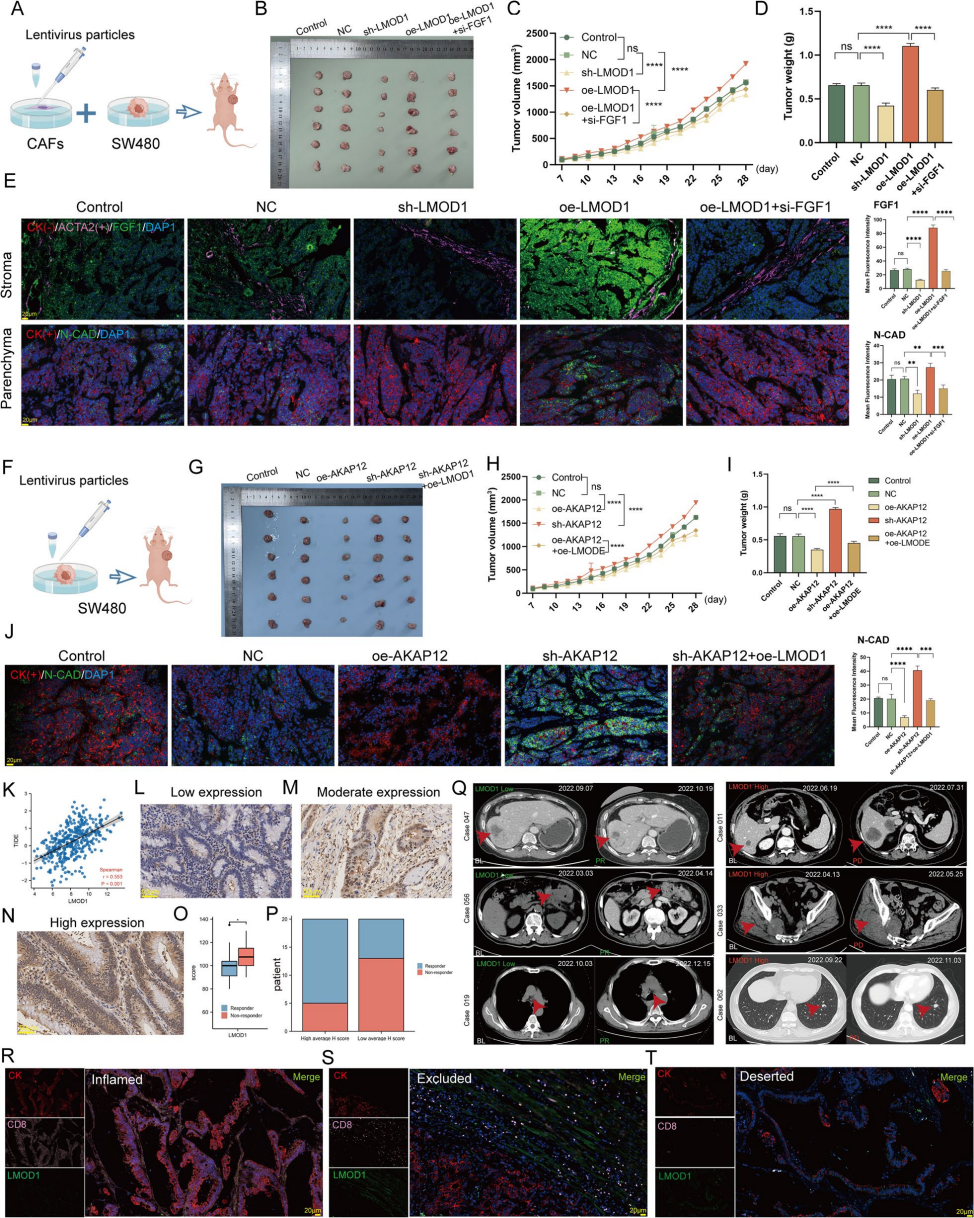
In vivo experiment to validate the mechanism of LMOD1. **A** Diagram of the animal experiments. **B** Mouse xenograft tumors (n = 6 mice/group). **C** Xenograft tumor volumes. **D** Xenograft tumor’s weights at the end of the investigation. **E** mIF staining of FGF1 (green) and N-cadherin (green) proteins in mouse xenograft tumor stromal and parenchymal tissues. ACTA2 (pink), DAPI (Blue) and CK (red) for tissue-localization (Magnification, × 400, scale bars = 20 μm). **F** The diagram showing the procedures used in animal experiments in vivo. **G** Mouse xenograft tumors (n = 6 mice/group). **H** Xenograft tumor volumes. **I** Xenograft tumor’s weights at the end of the investigation. **J** mIF staining of N-cadherin (green) proteins in mouse xenograft tumor parenchymal tissues. DAPI (Blue) and CK (red) for tissue-localization. (Magnification, × 400, scale bars = 20 μm). **K** The association of *LMOD1* mRNA expression with TIDE score as determined on the TCGA-CRC database (Spearman method, n = 620). **L**–**N** CRC tissue’s LMOD1 proteins IHC staining (Magnification, × 400, scale bars = 20 μm). **O** Bar plot showing the specific response rates for the high- and low-LMOD1 average H-score groups in 40 CRC patients. **P** Box plot illustrating the specific LMOD1 expression (H-Score) between non-responder and responder post anti-PD-1 therapy in 40 CRC patients (Wilcoxon test, *P < 0.05). **Q** Representative pictures of CT scan. Primary or metastatic tumor foci measured before initiation of immunotherapy (Baseline, BL). The red arrows indicate the primary or metastatic tumor foci. PD for progressive disease (PD), PR for partial response (PR). The expression level of LMOD1 can predict immunotherapy. **R**–**T** The co-stained LMOD1, CK, and CD8 images in the 3 immunophenotypes. Based on the spatial CD8 + T cells distribution, CRC tissues are categorized into 3 immunophenotypes, immune excluded, immune inflamed, and immune desert. Different sample measurements were taken. (Magnification, × 400, scale bars = 20 μm). Data are presented as the mean ± SEM ****P < 0.0001, ***P < 0.001, **P < 0.01, *P < 0.05. All experiments were repeated at least three times, independently

Subsequently, the biphasic function of LMOD1 was validated in vivo. BALB/c mice were subcutaneously co-injected with fibroblasts and cancer cells. To specifically analyze the role of CAF-derived LMOD1, primary CAFs were transduced with lentivirus particles to alter LMOD1 expression and then injected subcutaneously into BALB/c mice mixed with untreated SW480 (Fig. 8A). It was found that LMOD1 overexpression significantly promoted tumor growth; however, this effect was attenuated in mice carrying si-FGF1 tumors (Fig. 8B–D, P < 0.0001), confirming that LMOD1 stimulates tumor growth in vivo by regulating FGF1. Further, mIF staining confirmed that LMOD1 overexpression promoted FGF1 expression in mouse stromal tissue; however, this effect was not evident in parenchyma (Fig. 8E).

To specifically analyze the role of cancer cell-derived AKAP12/LMOD1, SW480 cells were transduced with lentivirus particles to alter AKAP12/LMOD1 expression and then injected subcutaneously into BALB/c mice (Fig. 8F). Results showed that AKAP12 interference significantly promoted tumor growth; however, this effect was attenuated in mice carrying oe-LMOD1 tumors (Fig. 8G–I, P < 0.0001), confirming that AKAP12 inhibits tumor growth in vivo by regulating LMOD1. mIF staining further confirmed that interference with AKAP12 reduced LMOD1 expression in mouse parenchyma tissue (Fig. 8J).

Continued activation of stromal CAFs promotes tumor fibrosis, which is an importantmalignant features of advanced tumors [57]. The production of collagen by CAFs improves the stiffness of the TME Matrigel matrix, creating a barrier that prevents the killing of cancer cells by cytotoxic T cells and promotes immune escape [58]. This stroma-restricted phenotype is referred to as an immune-exclusion tumor, which occurs in patients who may not benefit from immunotherapy and thus have poor prognosis [59]. Previous studies reported that LMOD1 is a positive regulator of CAFs activation (Fig. 5T, U). Based on previous TIDE studies, we extracted gene expression signatures linked to T cell exclusion and dysfunction. In TCGA-CRC, we observed a significant positive correlation between LMOD1 and TIDE score (Fig. 8K, R = 0.553, P < 0.001), indicating that LMOD1 can stimulate resistance to immunotherapy. Further, LMOD1 scores were assigned to each CRC patient in a small cohort (n = 40) based on the IHC staining of LMOD1 (Fig. 8L–N) to explore the relationship of LMOD1 expression with immunotherapy response. The inclusion/exclusion criteria are presented in the Supplemental material. It was found that responsive patients had lower LMOD1 expression than non-responder patients (Fig. 8O, P < 0.05). Patients with high LMOD1 expression exhibited lower response rates relative to those with high LMOD1 expression (Fig. 8P). Moreover, analysis of imaging data revealed that patients with a high LMOD1 expression experienced earlier progression (Fig. 8Q, red arrows represent metastatic foci). Thus, we classified all CRC samples into three immune subtypes: immune-inflamed, immune-excluded, and immune-desert tumors. Notably, most tumors with high LMOD1 expression were immune exclusion tumors, whereas those tumors with low LMOD1 expression were immune inflamed and immune desert tumors (Fig. 8R, T). This confirms our previous inference that LMOD1 can inhibit immune infiltration.

## Discussion

As with multicellular organisms, the basic functional unit of a tumor is the individual cell, which sends and receives signals from its neighbors to maintain the malignant character of cancer cells [60]. Cell-to-cell communication during this process is a key mechanism leading to TME heterogeneity [61]. In particular, specialized cell surface protein complexes (TJs and GJs) which form cellular junctions contribute to the cellular heterogeneity during tumor evolution [62]. For instance, at the beginning of the EMT procedure (for epithelial cells), TJs are deconstructed, tight intercellular contacts are disrupted, and TJs proteins are relocated and/or degraded [63]. Thus, epithelial cells lose the normal intercommunication and mutual inhibition, leading to the transformation of cells into invasive cells and disseminating to distant sites [64]. This investigation revealed that TJs were significantly weakened during the transformation of intestinal epithelial cells into CRC cells. In addition, the expression of TJs proteins was decreased (which also manifested itself as a decrease in the number of TJs), and the structure of the TJs also became looser. This indicated that TJs have oncogenic roles during intestinal epithelial malignancy. Recent studies have shown that ZEB1 inhibits claudin-1 expression in CRC thereby, promoting cell invasion and metastasis [65].

In our previous study, we demonstrated that GJs proteins are rarely expressed in normal intestinal stroma but their expression is upregulated in the tumor stroma [44]. Therefore, we further explored the significance of GJs in the malignant transformation of stromal components. Similar to our previous findings, we found that GJs is enhanced in malignant stroma. Under physiological conditions, fibroblasts do not differentiate into an activated state following the alterations in GJs proteins 43 (Cx43) and 26 (Cx26), while an increase in Cx43 expression is accompanied by an increase in GJs function [66–68]. Under pathological conditions (e.g., tumors), myofibroblasts and fibroblasts maintain the metabolic acid load of cancer cells by providing the molecular apparatus that facilitates excretion of acid by tumor cells (via GJs) [69, 70]. The tumor mesenchyme plays a unique and critical role in transferring excess H + ions away from cancer cells, thereby protecting the TME. This process relies heavily on well-formed GJ connections between CAFs to ensure efficient communication [10]. The “seed and soil” theory proposes that cancer cells (“seeds”) can only grow in the appropriate conditions (“soil”) [71]. The “soil”, the environment in which cancer cells grow, consists of the extracellular matrix (ECM) and various cells in TME [72]. Fibroblasts secrete the major ECM components, and proteases which remodel the structure of the ECM. Studies have shown that dense collagen arrangement and high ECM stiffness can create a malignant phenotype in epithelial cells [73]. Therefore, the tumor stroma, represented by CAFs, is largely a carcinogenic component.

Based on aforementioned properties and functions of GJs and TJs, we defined two types of CRC communication phenotypes in the TCGA data. Significant differences in clinical features, biological signatures, and levels of immune cell infiltration were recorded between different clusters. In this case, cluster with poorer prognosis (C2) exhibited enhanced GJs signature (and a weak TJs signature), while the cluster with better prognosis (C1) had a stronger TJs signature (and a weaker GJs signature). Our findings, combined with existing research, suggest that the stroma in C2 tumors may be more aggressive, while the epithelial tissue in C1 tumors could be less malignant. This theory was later supported by enrichment analysis and analysis of pathological sections. Studies have clearly demonstrated that differences in the mRNA transcriptome between different CC subtypes correlate significantly with signaling pathways related to collagen and matrix formation. We also found that C2 has higher stromal abundance and lower tumor purity, both of which are associated with poorer tumor prognosis and often lead to poor immunotherapy response. Considering that the success of ICB depends on CD8 + T cells infiltration into the tumor, as well as the ability of the stroma to inhibit CD8 + T cells from approaching the cancer cells (an immune-exclusion effect), stromal depletion may be an effective strategy to improve response to immunotherapy. This study also found that C2 had a higher TIDE score (higher scores indicate more difficulty in benefiting from immunotherapy).

Given the high heterogeneity of CC phenotypes in CRC patients, it imperative to search for potential regulators of the CC phenotype to develop new combination therapy strategies and immunotherapeutic agents. Finally, a gene co-expression network (MEGENA) was constructed and a COX model was established. Analysis of the showed that LMOD1 was an important regulator. Notably, LMOD1 expression was found positively associated with the clinical stage of the tumor. LMOD1 exhibits a unique biphasic role, resembling TJs in epithelial cells, acting as a potential oncogene, and GJs in fibroblasts, exerting a pro-oncogenic function. On the one hand, LMOD1 regulates the expression of FGF1 to promote fibroblast activation and complete the EMT process of cancer cells in a fibroblast-dependent manner. It has also been shown to be modulated by AKAP12, which directly inhibits EMT in cancer cells. FGF1 was overexpressed in tumors compared to the paracancerous tissues [74]. Numerous studies have shown that FGF1 promotes tumor conformity and is positively correlated with mesenchymal phenotype [75, 76]. As for AKAP12, a newly identified oncogenic factor, the RBMS1-AKAP12 regulatory axis inhibited EMT and liver metastasis in a CRC progression model [77]. The different signaling pathways triggered by LMOD1 in the tumor's tissue (parenchyma) and surrounding connective tissue (stroma) exemplify the concept of tumor heterogeneity. This phenomenon, where a single molecule exhibits variations in expression based on location and time, often reflects intricate sequences of molecular signaling, ultimately leading to diverse cell populations within the tumor. As previously described, tumor ECM contributes to drug resistance and immunosuppression [78]. Increased collagen production is associated with depletion of CD8 + T cell subsets [79]. Recent studies have shown that LMOD1 exerts effect of CAFs in 5-FU resistance [80]. This molecular mechanism promotes immune escape from tumors, but its role in immunophenotyping and immune infiltration in CRC is not well understood. We have verified the effect of LMOD1 on immune infiltration and immunophenotyping of CRC through multiple experiments. Tumors overexpressing LMOD1 are immune-excluded and patients with high expression of LMOD1 have a poor response to immunotherapy, suggesting that targeting LMOD1 may enhance the efficacy of immunotherapy for CRC.

We acknowledge that our study has some limitations. Although we distinguished between TJs and GJs in epithelial cells and fibroblasts, respectively, we cannot rule out the possibility of internal heterogeneity among TJs and GJs themselves, and this heterogeneity could not be determined due to the limited number of sequenced samples. We are enrolling patients for a multicentre clinical cohort to further analyze and validate our findings. The clinical typing in this study was based on publicly available data, and further large-scale protein sequencing analyzes are needed to test the effectiveness of this stratification strategy in the clinic. Our findings indicate that LMOD1 plays a multifaceted role in CRC progression. However, the specific mechanisms by which its key oncogenic functions contribute to this process and how they connect to cellular communication remain unclear. We are conducting further investigations to address these knowledge gaps.

## Conclusion

There is heterogeneity in the expression of CC molecules in the development of CRC, and this heterogeneity is both spatial and temporal. The CC-related gene *LMOD1* exhibits a biphasic function, providing a molecular basis for further understanding of TME heterogeneity.

### Supplementary Information

Additional file 1.

## Data Availability

The data and materials in the current study are available from the corresponding author: Yu-gen Chen: chenyugen888@126.com.

## Abbreviations

CC: Cellular communication
TJs: Tight junctions
GJs: Gap junctions
TME: Tumor microenvironment
CRC: Colorectal cancer
LMOD1: Leiomodin1
EMT: Epithelial–mesenchymal transition
TILs: Tumor infiltrating lymphocytes
ICBs: Immune checkpoint inhibitors
TCGA: The Cancer Genome Atlas
UCSC: University of California Santa Cruz
TPM: Transcripts Per kilobase Million
ST: Spatial transcriptome
OS: Overall survival
PFI: Progression-free interval
DSS: Disease-specific survival
HUGO: Human Genome Organization
DEGs: Differentially Expressed Genes
MEGENA: Multiscale embedded gene co-expression network analysis
PFN: Planar filtered network
MCA: Multiscale clustering analysis
TIDE: Tumor immune dysfunction and exclusion
PBS: Phosphate-buffered saline
DNase: Deoxyribonuclease
BSA: Bovine serum albumin
DMEM: Dulbecco’s modified Eagles medium
PCs: Principal components
UMAP: Uniform manifold approximation and projection
HE: Hematoxylin and eosin
IHC: Immunohistochemical
TSA: Tyramide signal amplification
HIER: Heating-induced epitope retrieval
FBS: Fetal bovine serum
WB: Western blotting
RIPA: Radioimmunoprecipitation
SDS-PAGE: Sodium dodecyl-sulfate polyacrylamide
PVDF: Polyvinylidene fluoride
AVMA: American Veterinary Medical Association
GSEA: Gene set enrichment analysis
FGFs: Fibroblast growth factors
Cx43: GJs proteins 43
ECM: Extracellular matrix
