# Supplementary material for: Spatiotemporal heterogeneity of LMOD1 expression summarizes two modes of cell communication in colorectal cancer
Source: J Transl Med. 2024 Jun 7;22:549. doi: 10.1186/s12967-024-05369-3 (PMC11161970; doi:10.1186/s12967-024-05369-3)
Supplement: Supplementary file 1 — Additional file 1. [file 12967_2024_5369_MOESM1_ESM.docx]

**Supplemental Methods**

## Table S1

**Table S1. All the antibodies and reagents utilized in this investigation**

| Antibodies and Reagents | Manufacturer, Country, Cat number, Lot number | Concentration |
| --- | --- | --- |
| Roswell Park Memorial Institute 1640 (RPMI-1640) | Gibco, USA, 31870082, 8123063 | - |
| Dulbecco’s modified Eagle’s medium (DMEM) | Gibco, USA, C11995500BT, 8122778 | - |
| Phosphate buffered saline (PBS) | Gibco, USA, 70011-044, 8123148 | - |
| Fetal bovine serum (FBS) | Gibco, USA, 10099-141, 2375386CP | - |
| Trypsin | Gibco, USA, 25300-054, 2509042 | - |
| Matrigel | Corning, USA, 356234, 1074003 | - |
| Puromycin | Beyotime Biotechnology, China，ST551-10mg,050823230613 | - |
| 4% Paraformaldehyde | Biosharp, China, BL539A,23159313 |  |
| 4',6-diamidino-2-phenylindole  (DAPI) | Beyotime Biotechnology, China, C1002,  091620210520 | - |
| Immunastaining Permeabilization Buffer with TritonX-100 | Beyotime Biotechnology, China, P0096-100ml,042921211027 |  |
| Protease and phosphatase inhibitor cocktail for genneraluse,50X | Beyotime Biotechnology, China, P1045,051823230618 |  |
| RBC Lysis Buffer (10×) | Biosharp, China, CS003,220903 |  |
| bovine serum albumin (BSA) | Vazyme, USA, B2270DBA,027E2270DA |  |
| Collagenase III | Biosharp, China, BS164-100mg, B0013K030100 |  |
| Deoxyribonuclease I | Beyotime Biotechnology, China, D7073, 112522230619 |  |
| Crystal violet | Beyotime Biotechnology, China, C0121-100ml,121322230524 |  |
| Radioimmunoprecipitation assay buffer (RIPA buffer) | Beyotime Biotechnology,China，P0013B,052523230703 |  |
| Sodium dodecyl-sulfate polyacrylamide gel electrophoresis (SDS-PAGE) | EpiZyme, China, LK209,02652030 |  |
| Tricolor Prestained Protein Marker | EpiZyme, China, WJ103,027352000 |  |
| FGF1small interfering (si) RNA | Corues Biotechnology, China,1001CR01, 202304 |  |
| Lipofectamine 3000 | Invitrogen, USA, L3000-015, 2395298 | - |
| Opti-MEM | Gibco, USA, 31985-070, 2276923 | - |
| Bicinchoninic acid (BCA)  Assay Kit | Thermo Fisher Scientific, USA, 23227, XJ357433 | - |
| Tyramide signal amplification biotin system kit | UElady Biotechnology, China, Y6082L,210413L2-1 | - |
| Hematoxylin-Eosin staining Kit | Solarbio life sciences, China, G1120, 20220325 | - |
| α-Smooth Muscle Actin (D4K9N) | Cell Signaling Technology, USA, 19245S, 3 | IF: 1:320 |
| FAP (E1V9V) Rabbit mAb | Cell Signaling Technology, USA, 66562, 5 | IF: 1:100 |
| Anti-CK Rabbit polyclonal Antibody | Cell Signaling Technology, USA, 12509, 3 | WB 1:1000 |
| LMOD1 Polyclonal Antibody | Thermo Fisher Scientific, USA, PA5-55848, 000024470 | WB: 0.4 µg/mL  IF: 1:100 |
| AKAP12 Polyclonal Antibody | Thermo Fisher Scientific, USA, PA5-21759, YF3971900A | WB: 1:1000 |
| Anti-GJA4 Polyclonal Antibody | Solarbio life sciences, China, K003394P，209G037 | IF 1:200 |
| Claudin 4 Polyclonal Antibody | Thermo Fisher Scientific, USA, 36-4800, YC371872 | IF 1:200 |
| EpCAM Recombinant Rabbit Monoclonal Antibody | Thermo Fisher Scientific, USA, MA5-29246, YF3956931 | IF 1:200 |
| Rabbit polyclonal antibody to FGF1 | Affinity Biosciences, China, DF6124,16b3122 | WB: 1:1000 |
| Anti-β-actin Mouse polyclonal Antibody | Proteintech, USA, 20536-1-AP, 81115-1-RR | WB 1:5000 |
| Anti-GAPDH Mouse polyclonal Antibody | Abbrab, China, AB300016, 4659180001 | WB 1:3000 |
| Anti-N-cadherin Rabbit polyclonal Antibody | Cell Signaling Technology, USA, 4061, 12 | WB 1:1000 |
| Anti-E-cadherin Rabbit polyclonal Antibody | Cell Signaling Technology, USA, 3195, 6 | WB 1:1000 |
| Anti-MMP9 Rabbit polyclonal Antibody | Cell Signaling Technology, USA, 13667, 5 | WB 1:1000 |
| Anti-MMP2 Rabbit polyclonal Antibody | Cell Signaling Technology, USA, 40994, 3 | WB 1:1000 |
| Anti-Snail Rabbit polyclonal | Cell Signaling Technology, USA, 3879, 6 | WB 1:1000 |
| Anti-Slug Rabbit polyclonal | Cell Signaling Technology, USA, 9585, 12 | WB 1:1000 |
| Anti-CD8 Mouse polyclonal Antibody | Cell Signaling Technology, USA, 55397, 3 | WB 1:1000  IF 1:400 |
| CoraLite488-conjugated Goat Anti-Rabbit IgG(H+L) | Proteintech, USA, SA00013-2, 205001014 | IF 1:300 |
| CoraLite594-conjugated Goat Anti-Mouse IgG(H+L) | Proteintech, USA, SA00013-3, 20000154 | IF 1:300 |
| CoraLite647-conjugated Mouse Anti-Heavy Chain of Rabbit IgG | Proteintech, USA, SA00014-6, 20000129 | IF 1:300 |
| Highly Cross-Adsorbed Goat (Polyclonal) Anti-Mouse IgG(H+L) Antibody | LI-COR, USA, 926-68070, Q04695 | WB 1:5000 |
| Highly Cross-Adsorbed Goat (Polyclonal) Anti-Rabbit IgG(H+L) Antibody | LI-COR, USA, 926-68071, S11385 | WB 1:5000 |

## Table S2

**Table S2. Human Genome Organization (HUGO) portal on molecules of the tight junctions (TJs) and gap junctions (GJs)**

## Figure S1

**
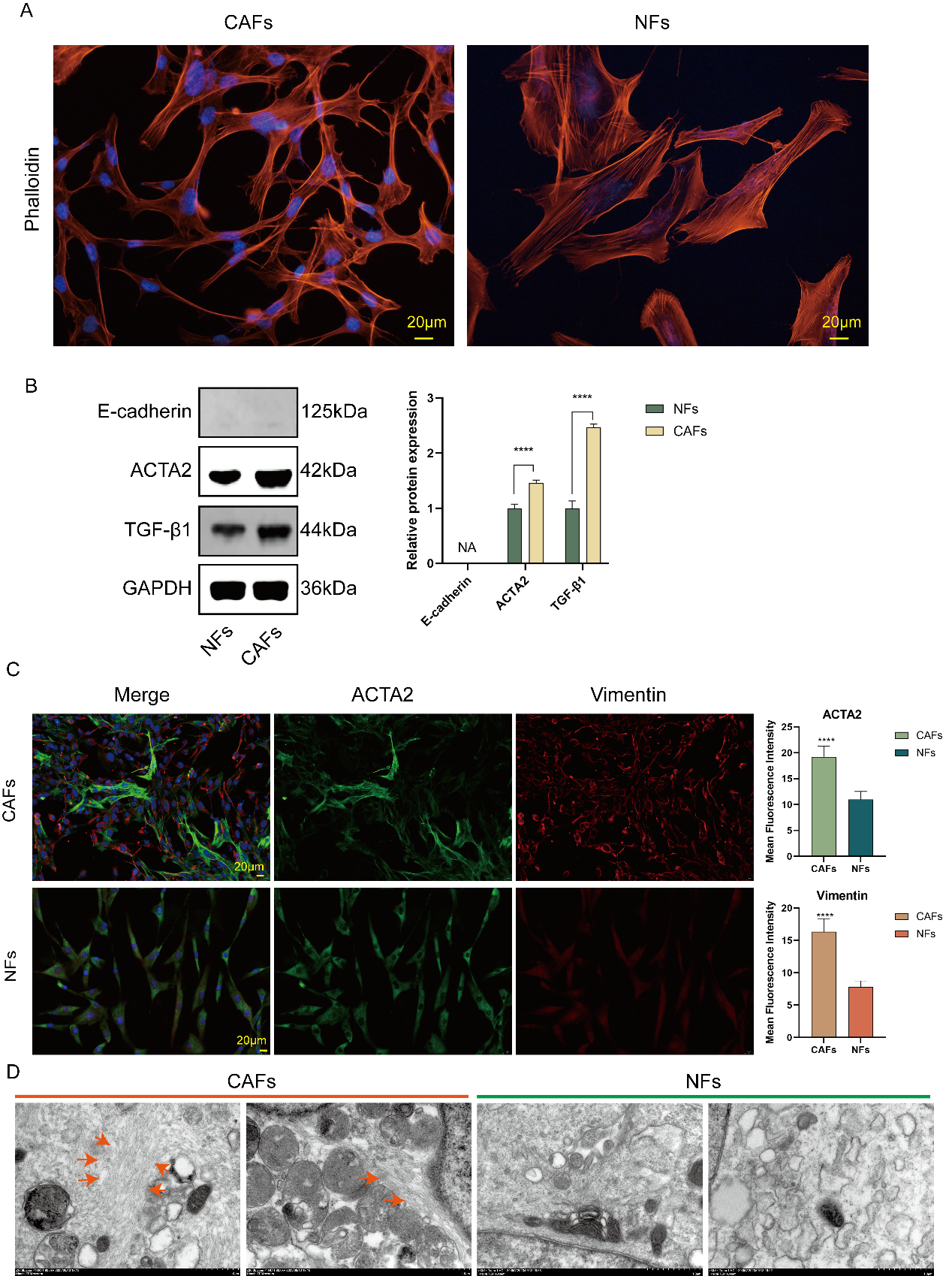
**

**Figure S1. Isolation and characterization of primary Fibroblasts.**

(A) Primary CAFs and NFs were isolated from patients with CRC. CAFs and NFs were stained with phalloidin and DAPI to visualize the actin cytoskeleton and nuclei, respectively. CAFs were shuttle, star or polygonal in shape, scattered and irregularly arranged, with a more obvious actin skeleton visible in the cytoplasm. Morphologically, NFs were long pike-shaped and grew in bundles, weaves, and vortexes. (B) Western blot results showed that Neither CAFs nor NFs expressed E-cadherin, and the expression levels of TGF-β1 and ACTA2 were increased in CAFs compared to NFs. (C) Immunofluorescence showed that ACTA2 was arranged in parallel and regularly in NFs, but not in CAFs, and the expression level of Vimentin, ACTA2 was higher than that in NFs. (D) Electron microscopy showed that dense, regularly arranged myofilaments were visible in the cytoplasm of CAFs (orange arrow) but not in NFs. Data are expressed as mean ± SEM, ****p< 0.0001. All experiments were repeated at least three times, independently.

## Gene Perturbation

### LMOD1 shRNA and overexpression preparation

**Table S3. Gene information**

| **Gene symbol** | **GenBank_ID** |
| --- | --- |
| *LMOD1* | NM_012134.3 |

**Table S4. Target information**

| **NO.** | **Accession** | **Target Seq** | **CDS** | **GC%** |
| --- | --- | --- | --- | --- |
| LMOD1-RNAi (123235-1) | NM_012134 | CTCTCCAAAGAACTCACCCAA | 209..2011 | 47.37% |
| LMOD1-RNAi (123236-1) | NM_012134 | CGTCAACAACTCAGACTGCAT | 209..2011 | 42.11% |
| LMOD1-RNAi (123237-11) | NM_012134 | CCCAGCATATTTGATGAGCCT | 209..2011 | 42.11% |

**Plasmid name：**GV493

**Negative control insert sequence：TTCTCCGAACGTGTCACGT**

**Table S5. Synthetic oligo information**

| **NO.** | **5’** | **STEM** | **Loop** | **STEM** | **3’** |
| --- | --- | --- | --- | --- | --- |
| LMOD1-RNAi(123235-1)-a | Ccgg | CTCTCCAAAGAACTCACCCAA | CTCGAG | TTGGGTGAGTTCTTTGGAGAG | TTTTTg |
| LMOD1-RNAi(123235-1)-b | aattcaaaaa | CTCTCCAAAGAACTCACCCAA | CTCGAG | TTGGGTGAGTTCTTTGGAGAG |  |
| LMOD1-RNAi(123236-1)-a | Ccgg | CGTCAACAACTCAGACTGCAT | CTCGAG | ATGCAGTCTGAGTTGTTGACG | TTTTTg |
| LMOD1-RNAi(123236-1)-b | aattcaaaaa | CGTCAACAACTCAGACTGCAT | CTCGAG | ATGCAGTCTGAGTTGTTGACG |  |
| LMOD1-RNAi(123237-11)-a | Ccgg | CCCAGCATATTTGATGAGCCT | CTCGAG | AGGCTCATCAAATATGCTGGG | TTTTTg |
| LMOD1-RNAi(123237-11)-b | aattcaaaaa | CCCAGCATATTTGATGAGCCT | CTCGAG | AGGCTCATCAAATATGCTGGG |  |

**Table S6. Overexpression Plasmid information**

| **ID** | **seq** |
| --- | --- |
| LMOD1(93990-1)-p1 | AGGTCGACTCTAGAGGATCCCGCCACCATGTCTAGAGTAGCCAAATATC |
| LMOD1(93990-1)-p2 | TCCTTGTAGTCCATACCCTGAAGCAGTTTGGGCACTTCCAC |

**Plasmid name：**GV492

**The positive clones sequencing results were analyzed**

**The comparison results were shown as follows：**

CGAAGCTTGGGCTGCAGGTCGACTCTAGAGGATCCCGCCACCATGTCTAGAGTAGCCAAATATCGCCGGCAGGTGAGTGAAGACCCCGACATCGACAGCCTGCTGGAGACCCTGTCTCCCGAGGAGATGGAGGAGCTGGAGAAGGAGCTGGACGTGGTGGACCCAGACGGGAGTGTTCCCGTGGGGCTGCGGCAGAGAAACCAGACGGAGAAACAGTCCACGGGTGTGTACAACCGGGAGGCCATGCTCAACTTCTGTGAAAAGGAGACCAAGAAACTTATGCAGAGGGAGATGTCCATGGATGAAAGCAAGCAAGTGGAGACCAAGACAGATGCCAAGAATGGAGAGGAAAGGGGCAGAGATGCCAGCAAAAAAGCCCTGGGCCCCAGACGGGACTCAGATCTGGGGAAGGAGCCAAAGAGGGGTGGTTTAAAGAAAAGCTTCTCTAGAGACAGAGATGAAGCTGGTGGCAAGAGTGGCGAGAAGCCCAAGGAGGAGAAGATCATCCGGGGCATTGACAAGGGCCGGGTCAGGGCTGCAGTGGATAAGAAGGAGGCAGGGAAGGATGGGAGAGGAGAGGAGAGGGCAGTGGCCACCAAGAAGGAAGAGGAGAAGAAAGGGAGTGACAGGAACACAGGCTTGAGCAGGGACAAGGATAAAAAGAGAGAGGAGATGAAGGAGGTGGCCAAGAAAGAGGATGATGAGAAGGTAAAAGGGGAGCGTAGGAACACAGACACCAGAAAAGAGGGTGAGAAGATGAAAAGAGCAGGTGGGAACACAGACATGAAAAAGGAGGATGAGAAGGTAAAAAGAGGAACTGGGAACACAGACACCAAAAAGGACGATGAAAAAGTCAAGAAGAATGAACCCTTACATGAAAAGGAAGCCAAGGATGACAGCAAGACCAAAACACCCGAGAAACAGACGCCCAGTGGCCCCACCAAGCCCTCTGAAGGACCGGCCAAGGTGGAGGAGGAGGCAGCTCCCAGCATATTTGATGAGCCTCTGGAGAGAGTGAAGAACAATGACCCCGAGATGACTGAGGTGAACGTCAACAACTCAGACTGCATCACAAATGAGATCTTGGTCCGGTTTACTGAGGCTCTGGAGTTCAACACTGTGGTTAAGCTGTTCGCCTTGGCCAACACGCGAGCCGATGACCACGTGGCCTTTGCCATTGCCATCATGCTCAAGGCCAACAAGACCATCACCAGCCTCAACCTGGACTCCAACCACATCACAGGCAAAGGCATCCTGGCCATCTTCCGGGCCCTCCTCCAGAACAACACGCTGACCGAGCTCCGCTTCCACAACCAGCGACACATCTGTGGAGGCAAGACGGAGATGGAGATCGCCAAGCTGCTGAAGGAGAATACTACCCTGCTCAAGCTGGGCTACCATTTTGAGCTGGCCGGGCCCCGAATGACTGTCACCAATCTGCTCAGCCGCAACATGGACAAGCAGAGACAAAAGCGGCTGCAGGAGCAAAGGCAGGCACAGGAAGCCAAGGGAGAGAAGAAGGATCTGCTGGAGGTACCCAAGGCCGGGGCCGTGGCTAAGGGCTCCCCAAAACCTTCACCTCAACCATCTCCAAAGCCCTCTCCAAAGAACTCACCCAAAAAAGGGGGTGCTCCAGCTGCCCCACCACCCCCTCCCCCTCCCTTGGCTCCACCCCTTATCATGGAGAACCTGAAGAATTCACTCTCACCAGCTACCCAGAGGAAGATGGGAGACAAAGTCCTCCCTGCCCAGGAGAAGAACTCCCGTGACCAGCTATTGGCTGCCATCCGCTCCAGCAACCTCAAGCAGCTCAAGAAGGTGGAAGTGCCCAAACTGCTTCAGGGTATGGACTACAAGGATGACGATGACAAGGATTACAAAGACGACGATGATAAGGACTATAAGGATGATGACGACAAATGAGCTAGCACAT

### AKAP12 shRNA and overexpression preparation

**Table S7. Gene information**

| **Gene symbol** | **GenBank_ID** |
| --- | --- |
| AKAP12 | NM_005100 |

**Table S8. Target information**

| **ID** | **Target Seq** | **Start position information** | **GC (%)** |
| --- | --- | --- | --- |
| AKAP12-RNAi(124718-1) | GCTACTACCAAGAAAGGCTTA | 4657 | 36.84 |
| AKAP12-RNAi(124719-1) | GCAAGGTGGATACCTCAGTAT | 2201 | 42.11 |
| AKAP12-RNAi(124720-1) | CCAGGCTAATGATATTGGATT | 684 | 31.58 |

**Table S9 Synthetic oligo information**

| **ID** | **5’** | **stem** | **loop** | **stem** | **3’** |
| --- | --- | --- | --- | --- | --- |
| AKAP12-RNAi(124718-1)-a | Ccgg | GCTACTACCAAGAAAGGCTTA | CTCGAG | TAAGCCTTTCTTGGTAGTAGC | TTTTTg |
| AKAP12-RNAi(124718-1)-b | aattcaaaaa | GCTACTACCAAGAAAGGCTTA | CTCGAG | TAAGCCTTTCTTGGTAGTAGC |  |
| AKAP12-RNAi(124719-1)-a | Ccgg | GCAAGGTGGATACCTCAGTAT | CTCGAG | ATACTGAGGTATCCACCTTGC | TTTTTg |
| AKAP12-RNAi(124719-1)-b | aattcaaaaa | GCAAGGTGGATACCTCAGTAT | CTCGAG | ATACTGAGGTATCCACCTTGC |  |
| AKAP12-RNAi(124720-1)-a | Ccgg | CCAGGCTAATGATATTGGATT | CTCGAG | AATCCAATATCATTAGCCTGG | TTTTTg |
| AKAP12-RNAi(124720-1)-b | aattcaaaaa | CCAGGCTAATGATATTGGATT | CTCGAG | AATCCAATATCATTAGCCTGG |  |

**Plasmid name：GV654**

**Negative control insert sequence：TTCTCCGAACGTGTCACGT**

**Table S10. Overexpression Plasmid information**

| **ID** | **seq** |
| --- | --- |
| AKAP12(95570-1)-p1 | CACACTGGACTAGTGGATCCCGCCACCATGGGCGCCGGGAGCTCCACCGAGCAG |
| AKAP12(95570-1)-p2 | AGTCACTTAAGCTTGGTACCGAAGATTCTGTAAGTTCTGACTTTGCAGATTC |

**Plasmid name：**GV657

**Control number：**CON468

**The positive clones sequencing results were analyzed**

**The comparison results were shown as follows：**

CGGCCGCCACTGTGCTGGATATCTGCAGAATTCCACCACACTGGACTAGTGGATCCCGCCACCATGGGCGCCGGGAGCTCCACCGAGCAGCGCAGCCCGGAGCAGCCGCCCGAGGGGAGCTCCACGCCGGCTGAGCCCGAGCCCAGCGGCGGCGGCCCCTCGGCCGAGGCGGCGCCAGACACCACCGCGGACCCCGCCATCGCTGCCTCGGACCCCGCCACCAAGCTCCTACAGAAGAATGGTCAGCTGTCCACCATCAATGGCGTAGCTGAGCAAGATGAGCTCAGCCTCCAGGAGGGTGACCTAAATGGCCAGAAAGGAGCCCTGAACGGTCAAGGAGCCCTAAACAGCCAGGAGGAAGAAGAAGTCATTGTCACAGAGGTTGGACAGAGAGACTCTGAAGATGTGAGCAAAAGAGACTCCGATAAAGAGATGGCTACTAAGTCAGCGGTTGTTCACGACATCACAGATGATGGGCAGGAGGAGACACCCGAAATAATCGAACAGATTCCTTCTTCAGAAAGCAATTTAGAAGAGCTAACACAACCCACTGAGTCCCAGGCTAATGATATTGGATTTAAGAAGGTGTTTAAGTTTGTTGGCTTTAAATTCACTGTGAAAAAGGATAAGACAGAGAAGCCTGACACTGTCCAGCTACTCACTGTGAAGAAAGATGAAGGGGAGGGAGCAGCAGGGGCTGGCGACCACAAGGACCCCAGCCTTGGGGCTGGAGAAGCAGCATCCAAAGAAAGCGAACCCAAACAATCTACAGAGAAACCCGAAGAGACCCTGAAGCGTGAGCAAAGCCACGCAGAAATTTCTCCCCCAGCCGAATCTGGCCAAGCAGTGGAGGAATGCAAAGAGGAAGGAGAAGAGAAACAAGAAAAAGAACCTAGCAAGTCTGCAGAATCTCCGACTAGTCCCGTGACCAGTGAAACAGGATCAACCTTCAAAAAATTCTTCACTCAAGGTTGGGCCGGCTGGCGCAAAAAGACCAGTTTCAGGAAGCCGAAGGAGGATGAAGTGGAAGCTTCAGAGAAGAAAAAGGAACAAGAGCCAGAAAAAGTAGACACAGAAGAAGACGGAAAGGCAGAGGTTGCCTCCGAGAAACTGACCGCCTCCGAGCAAGCCCACCCACAGGAGCCGGCAGAAAGTGCCCACGAGCCCCGGTTATCAGCTGAATATGAGAAAGTTGAGCTGCCCTCAGAGGAGCAAGTCAGTGGCTCGCAGGGACCTTCTGAAGAGAAACCTGCTCCGTTGGCGACAGAAGTGTTTGATGAGAAAATAGAAGTCCACCAAGAAGAGGTTGTGGCCGAAGTCCACGTCAGCACCGTGGAGGAGAGAACCGAAGAGCAGAAAACGGAGGTGGAAGAAACAGCAGGGTCTGTGCCAGCTGAAGAATTGGTTGAAATGGATGCAGAACCTCAGGAAGCTGAACCTGCCAAGGAGCTGGTGAAGCTCAAAGAAACGTGTGTTTCCGGAGAGGACCCTACACAGGGAGCTGACCTCAGTCCTGATGAGAAGGTGCTGTCCAAACCCCCCGAAGGCGTTGTGAGTGAGGTGGAAATGCTGTCATCACAGGAGAGAATGAAGGTGCAGGGAAGTCCACTAAAGAAGCTTTTTACCAGCACTGGCTTAAAAAAGCTTTCTGGAAAGAAACAGAAAGGGAAAAGAGGAGGAGGAGACGAGGAATCAGGGGAGCACACTCAGGTTCCAGCCGATTCTCCGGACAGCCAGGAGGAGCAAAAGGGCGAGAGCTCTGCCTCATCCCCTGAGGAGCCCGAGGAGATCACGTGTCTGGAAAAGGGCTTAGCCGAGGTGCAGCAGGATGGGGAAGCTGAAGAAGGAGCTACTTCCGATGGAGAGAAAAAAAGAGAAGGTGTCACTCCCTGGGCATCATTCAAAAAGATGGTGACGCCCAAGAAGCGTGTTAGACGGCCTTCGGAAAGTGATAAAGAAGATGAGCTGGACAAGGTCAAGAGCGCTACCTTGTCTTCCACCGAGAGCACAGCCTCTGAAATGCAAGAAGAAATGAAAGGGAGCGTGGAAGAGCCAAAGCCGGAAGAACCAAAGCGCAAGGTGGATACCTCAGTATCTTGGGAAGCTTTAATTTGTGTGGGATCATCCAAGAAAAGAGCAAGGAGAGGGTCCTCTTCTGATGAGGAAGGGGGACCAAAAGCAATGGGAGGAGACCACCAGAAAGCTGATGAGGCCGGAAAAGACAAAGAGACGGGGACAGACGGGATCCTTGCTGGTTCCCAAGAACATGATCCAGGGCAGGGAAGTTCCTCCCCGGAGCAAGCTGGAAGCCCTACCGAAGGGGAGGGCGTTTCCACCTGGGAGTCATTTAAAAGGTTAGTCACGCCAAGAAAAAAATCAAAGTCCAAGCTGGAAGAGAAAAGCGAAGACTCCATAGCTGGGTCTGGTGTAGAACATTCCACTCCAGACACTGAACCCGGTAAAGAAGAATCCTGGGTCTCAATCAAGAAGTTTATTCCTGGACGAAGGAAGAAAAGGCCAGATGGGAAACAAGAACAAGCCCCTGTTGAAGACGCAGGGCCAACAGGGGCCAACGAAGATGACTCTGATGTCCCGGCCGTGGTCCCTCTGTCTGAGTATGATGCTGTAGAAAGGGAGAAAATGGAGGCACAGCAAGCCCAAAAAAGCGCAGAGCAGCCCGAGCAGAAGGCAGCCACTGAGGTGTCCAAGGAGCTCAGCGAGAGTCAGGTTCATATGATGGCAGCAGCTGTCGCTGACGGGACGAGGGCAGCTACCATTATTGAAGAAAGGTCTCCTTCTTGGATATCTGCTTCAGTGACAGAACCTCTTGAACAAGTAGAAGCTGAAGCCGCACTGTTAACTGAGGAGGTATTGGAAAGAGAAGTAATTGCAGAAGAAGAACCCCCCACGGTTACTGAACCTCTGCCAGAGAACAGAGAGGCCCGGGGCGACACGGTCGTTAGTGAGGCGGAATTGACCCCCGAAGCTGTGACAGCTGCAGAAACTGCAGGGCCATTGGGTGCCGAAGAAGGAACCGAAGCATCTGCTGCTGAAGAGACCACAGAAATGGTGTCAGCAGTCTCCCAGTTAACCGACTCCCCAGACACCACAGAGGAGGCCACTCCGGTGCAGGAGGTGGAAGGTGGCGTACCTGACATAGAAGAGCAAGAGAGGCGGACTCAAGAGGTCCTCCAGGCAGTGGCAGAAAAAGTGAAAGAGGAATCCCAGCTGCCTGGCACCGGTGGGCCAGAAGATGTGCTTCAGCCTGTGCAGAGAGCAGAGGCAGAAAGACCAGAAGAGCAGGCTGAAGCGTCGGGTCTGAAGAAAGAGACGGATGTAGTGTTGAAAGTAGATGCTCAGGAGGCAAAAACTGAGCCTTTTACACAAGGGAAGGTGGTGGGGCAGACCACCCCAGAAAGCTTTGAAAAAGCTCCTCAAGTCACAGAGAGCATAGAGTCCAGTGAGCTTGTAACCACTTGTCAAGCCGAAACCTTAGCTGGGGTAAAATCACAGGAGATGGTGATGGAACAGGCTATCCCCCCTGACTCGGTGGAAACCCCTACAGACAGTGAGACTGATGGAAGCACCCCCGTAGCCGACTTTGACGCACCAGGCACAACCCAGAAAGACGAGATTGTGGAAATCCATGAGGAGAATGAGGTCGCATCTGGTACCCAGTCAGGGGGCACAGAAGCAGAGGCAGTTCCTGCACAGAAAGAGAGGCCTCCAGCACCTTCCAGTTTTGTGTTCCAGGAAGAAACTAAAGAACAATCAAAGATGGAAGACACTCTAGAGCATACAGATAAAGAGGTGTCAGTGGAAACTGTATCCATTCTGTCAAAGACTGAGGGGACTCAAGAGGCTGACCAGTATGCTGATGAGAAAACCAAAGACGTACCATTTTTCGAAGGACTTGAGGGGTCTATAGACACAGGCATAACAGTCAGTCGGGAAAAGGTCACTGAAGTTGCCCTTAAAGGTGAAGGGACAGAAGAAGCTGAATGTAAAAAGGATGATGCTCTTGAACTGCAGAGTCACGCTAAGTCTCCTCCATCCCCCGTGGAGAGAGAGATGGTAGTTCAAGTCGAAAGGGAGAAAACAGAAGCAGAGCCAACCCATGTGAATGAAGAGAAGCTTGAGCACGAAACAGCTGTTACCGTATCTGAAGAGGTCAGTAAGCAGCTCCTCCAGACAGTGAATGTGCCCATCATAGATGGGGCAAAGGAAGTCAGCAGTTTGGAAGGAAGCCCTCCTCCCTGCCTAGGTCAAGAGGAGGCAGTATGCACCAAAATTCAAGTTCAGAGCTCTGAGGCATCATTCACTCTAACAGCGGCTGCAGAGGAGGAAAAGGTCTTAGGAGAAACTGCCAACATTTTAGAAACAGGTGAAACGTTGGAGCCTGCAGGTGCACATTTAGTTCTGGAAGAGAAATCCTCTGAAAAAAATGAAGACTTTGCCGCTCATCCAGGGGAAGATGCTGTGCCCACAGGGCCCGACTGTCAGGCAAAATCGACACCAGTGATAGTATCTGCTACTACCAAGAAAGGCTTAAGTTCGGACCTGGAAGGAGAGAAAACCACATCACTGAAGTGGAAGTCAGATGAAGTCGATGAGCAGGTTGCTTGCCAGGAGGTCAAAGTGAGTGTAGCAATTGAGGATTTAGAGCCTGAAAATGGGATTTTGGAACTTGAGACCAAAAGCAGTAAACTTGTCCAAAACATCATCCAGACAGCCGTTGACCAGTTTGTACGTACAGAAGAAACAGCCACCGAAATGTTGACGTCTGAGTTACAGACACAAGCTCACGTGATAAAAGCTGACAGCCAGGACGCTGGACAGGAAACGGAGAAAGAAGGAGAGGAACCTCAGGCCTCTGCACAGGATGAAACACCAATTACTTCAGCCAAAGAGGAGTCAGAGTCAACCGCAGTGGGACAAGCACATTCTGATATTTCCAAAGACATGAGTGAAGCCTCAGAAAAGACCATGACTGTTGAGGTAGAAGGTTCCACTGTAAATGATCAGCAGCTGGAAGAGGTCGTCCTCCCATCTGAGGAAGAGGGAGGTGGAGCTGGAACAAAGTCTGTGCCAGAAGATGATGGTCATGCCTTGTTAGCAGAAAGAATAGAGAAGTCACTAGTTGAACCGAAAGAAGATGAAAAAGGTGATGATGTTGATGACCCTGAAAACCAGAACTCAGCCCTGGCTGATACTGATGCCTCAGGAGGCTTAACCAAAGAGTCCCCAGATACAAATGGACCAAAACAAAAAGAGAAGGAGGATGCCCAGGAAGTAGAATTGCAGGAAGGAAAAGTGCACAGTGAATCAGATAAAGCGATCACACCCCAAGCACAGGAGGAGTTACAGAAACAAGAGAGAGAATCTGCAAAGTCAGAACTTACAGAATCTTCGGTACCAAGCTTAAGTGACTACAAGGATGACGATGACAAGGATTACAAAGACGACGATGATAAGGACTATAAGGATGATGACGACAAATCTAGATAGTTAATTAAACCGGTAATAAAATATCTTTATTTTCATTACATCTGTGTGTTGGTTTTTTGTGTGAATCGATAGTACTAACATACGCTCTCCATCAAAACAAAACGAAACAAAACAAACTAGCAAAAT

The yellow fluorescent mark is：TCC synonymous mutation to TCG

### Figure S2

**
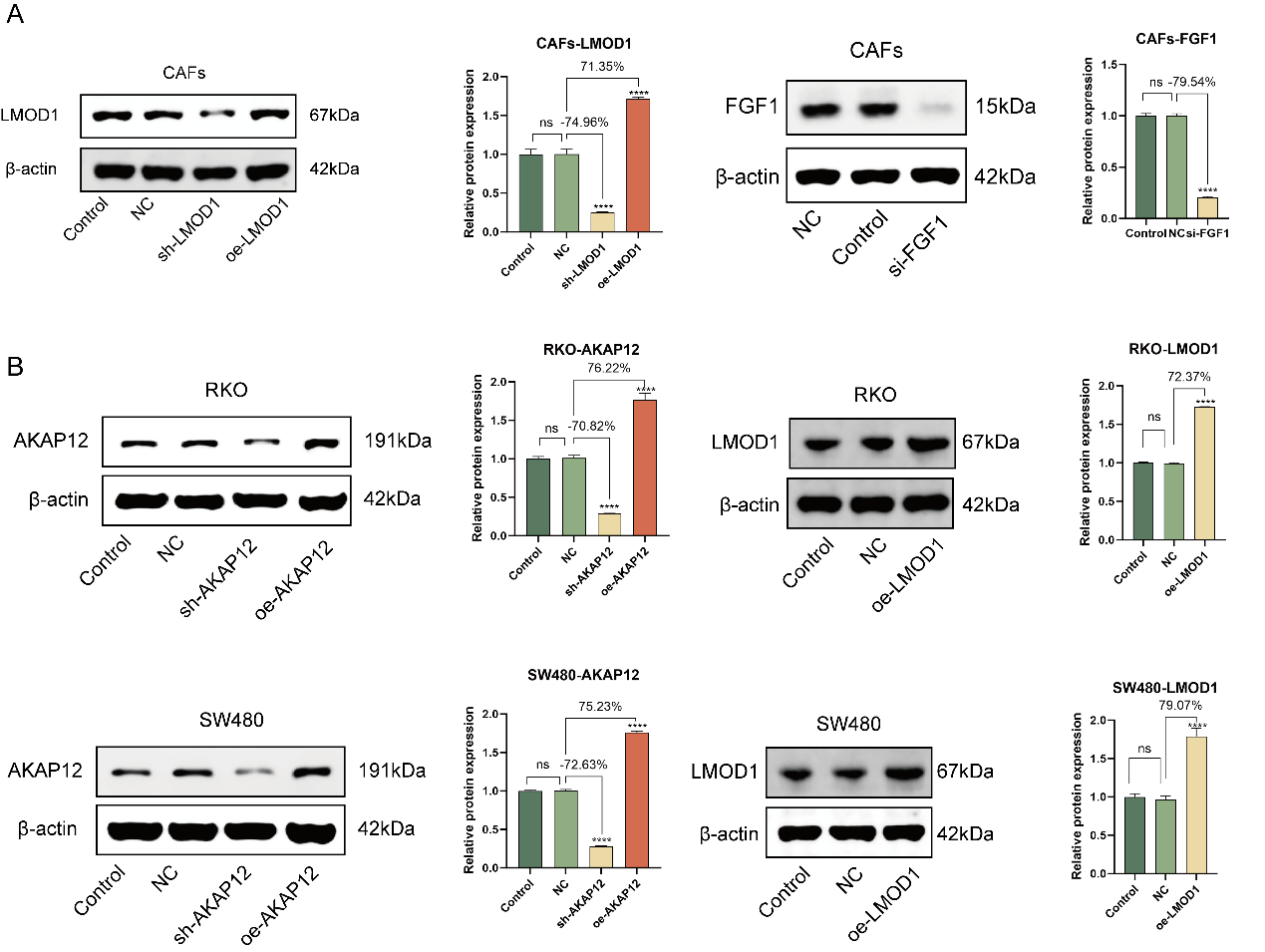
**

**Figure S2. Verification of transfection efficiency.**

(A-B) Transfection efficiency was verified by Western blot. Data are expressed as mean± SEM, **** P <0.0001. All experiments were repeated at least three times, independently.

## Inclusion/exclusion criteria for participants

This clinical retrospective observational study was carried out in accordance with the Declaration of Helsinki. The study’s protocol was approved by the ethics committee of the Jiangsu Province Hospital of Chinese Medicine (2020NL-107-01), and informed consent was obtained from clinicians and patients. This study initially enrolled 72 patients receiving pembrolizumab monotherapy for MSI-H/dMMR metastatic colorectal cancer (mCRC) in the oncology department of Jiangsu Province Hospital of Chinese Medicine (2022-01-01 and 2023-05-01). The decision regarding the immunotherapy timing and regimen was made by the treating physicians based on the current mCRC treatment guidelines. All patients had their diagnoses freshly confirmed and had not had any previous treatment. Each patient’s diagnosis was confirmed by clinical history, radiologic examination, and histopathology findings, they underwent endoscopy on admission and samples of tumor tissue were obtained by endoscopic biopsy. These patients received intravenous pabolizumab 200 mg every 3 weeks. In the end, 32 individuals were excluded from the analysis because they failed to fulfill the requirements for inclusion.

The International Union Against Cancer's recommended categories for tumor grade and stage were used[1]. Prior to analysis, all patient records and data were de-identified and anonymized. Inclusion criteria :(1) Clinical examination, enteroscopy, magnetic resonance imaging (MRI), PET-CT/CT, and hematological were used to diagnose all patients with mCRC for the first time. (2) Patient (age 18 -70) with mCRC who were admitted to the Jiangsu Province Hospital of Chinese Medicine between 2022-01-01 and 2023-05-01. Additionally, pathology samples from every patient were saved for two separate specialists to validate their re-diagnosis independently. (3) Complete clinical data were available for every patient. Immune checkpoint inhibitors were given to those patients until the illness worsened or the side effects became too bad. Every 6 weeks or as clinically necessary, a PET-CT or CT scan was used to review the patient's radiologic status. Evaluation Criteria in Solid Tumors, v1.1, was the basis for tumor response evaluation. The following patients were disqualified: (1) patients with a pathological diagnosis other than mCRC, such as intestinal stromal tumors; (2) patients who passed away during treatment; (3) patients with incomplete data or who lost to follow-up; (4) patients with an infection, an immune system disorder, a blood system condition; (5) patients who had local radiotherapy or radiofrequency ablation; (6) patients who were HIV positive; and (7) patients who were unable to tolerate adverse reactions.

**Supplemental Results**

## Supplemental Tables

### Table S11

**Table S11. Clinicopathological details of each patient**

### Table S12

**Table S12. GO and KEGG enrichment analyses of 157 DEGs between C1 and C2**

### Table S13

**Table S13. Module information**

| module.id | module. Size | module. Parent | module. Hub | module. *P* Value |
| --- | --- | --- | --- | --- |
| c1_2 | 33 | c1_1 | *COL1A2*(14) | 0 |
| c1_4 | 23 | c1_1 | *LMOD1*(12), *MYH11*(11) | 0 |
| c1_5 | 46 | c1_1 | *RAB31*(17), *GPNMB* (14) | 0 |
| c1_6 | 21 | c1_2 | *COL1A2*(12), *COL3A1*(11) | 0 |
| c1_11 | 27 | c1_5 | *RAB31*(14) | 0 |
| c1_12 | 19 | c1_5 | *CD163*(13) | 0 |
| c1_14 | 14 | c1_6 | *COL1A2*(12), *COL3A1*(10) | 0 |
| c1_16 | 16 | c1_11 | *RAB31*(12) | 0 |

## Supplemental Figures

### Figure S3


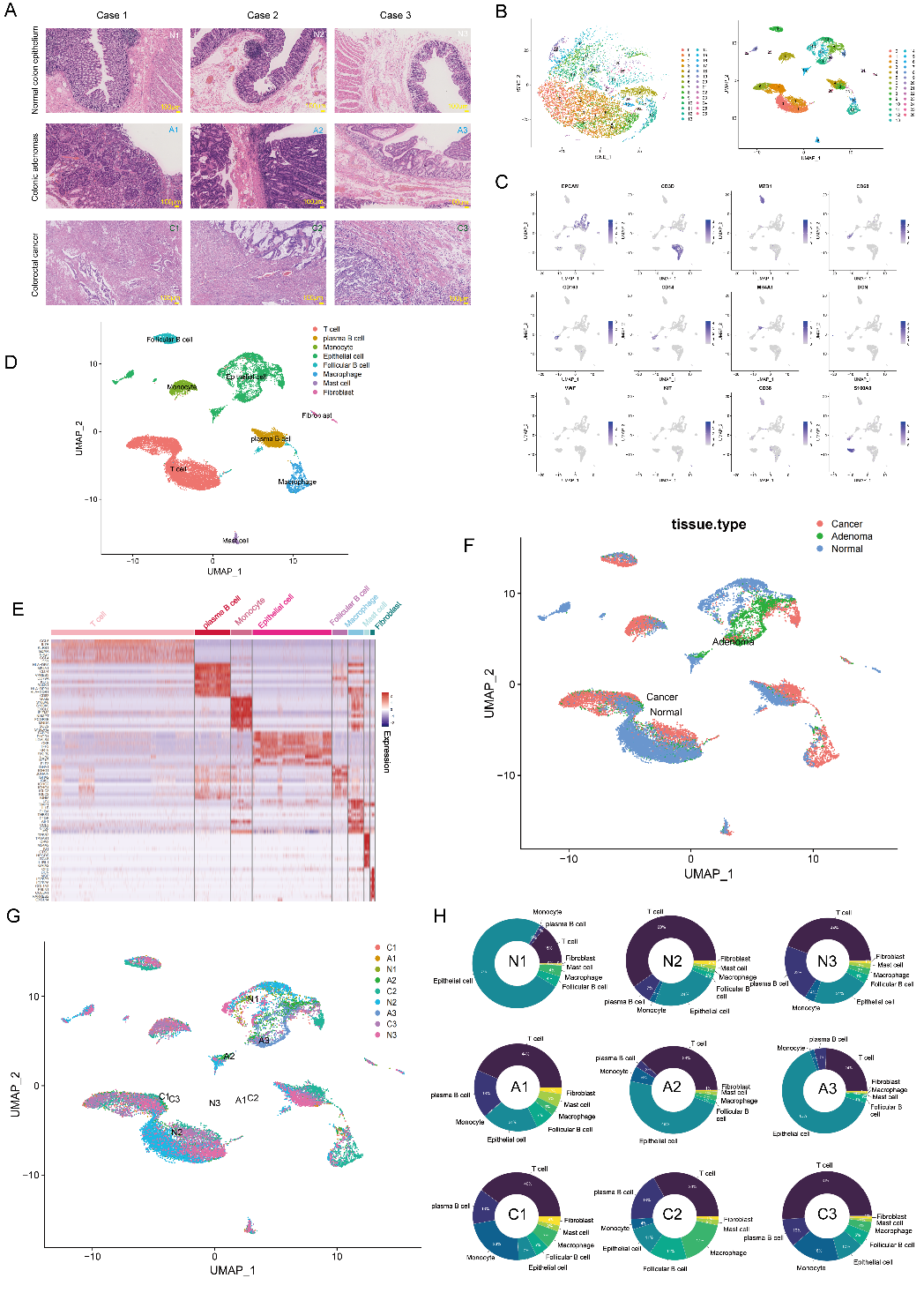


**Figure S3. Cellular atlas of normal colorectal, adenoma, and cancer tissue.**

(A) Hematoxylin and eosin (HE) staining of 9 samples in this study (scale bars =100 µm).

(B) The t-distributed Stochastic Neighbour Embedding (t-SNE) and Uniform Manifold Approximation and Projection (UMAP) were used for dimensionality reduction of the data.

(C) UMAP plot and violin plot show the marker genes from scRNA-seq used for cluster annotation. T cell (marked by CD3D), plasma B cell (marked by MS4A1), Monocyte (marked by S100A9), Epithelial cell (marked by EPCAM), Follicular B cell (marked by MZB1), Macrophage (marked by CD14, CD163, and CD68), plasma cells (marked by CD38), Endothelial cells (marked by VWF). Mast cell (marked by KIT), and Fiborblast (marked by DCN).

(D) The UMAP plot of 20,262 cells with high-quality to observe cell-type clusters on the basis of the well-known gene indices.

(E) Expression of ten representative genes with differential expression (relative expression level) for each cell type, shown using heatmap. Cell type-specfic genes were obtained with an unbiased fashion (Wilcoxon rank-sum test, FDR <0.01, and fold change >1.5).

(F-G) UAMP plots for the 20,262 high-quality cells showing tissue type (F) and sample origin (G).

(H) The proportion of each cell type in 9 samples. N: normal; A: adenoma; C: cancer.

### Figure S4


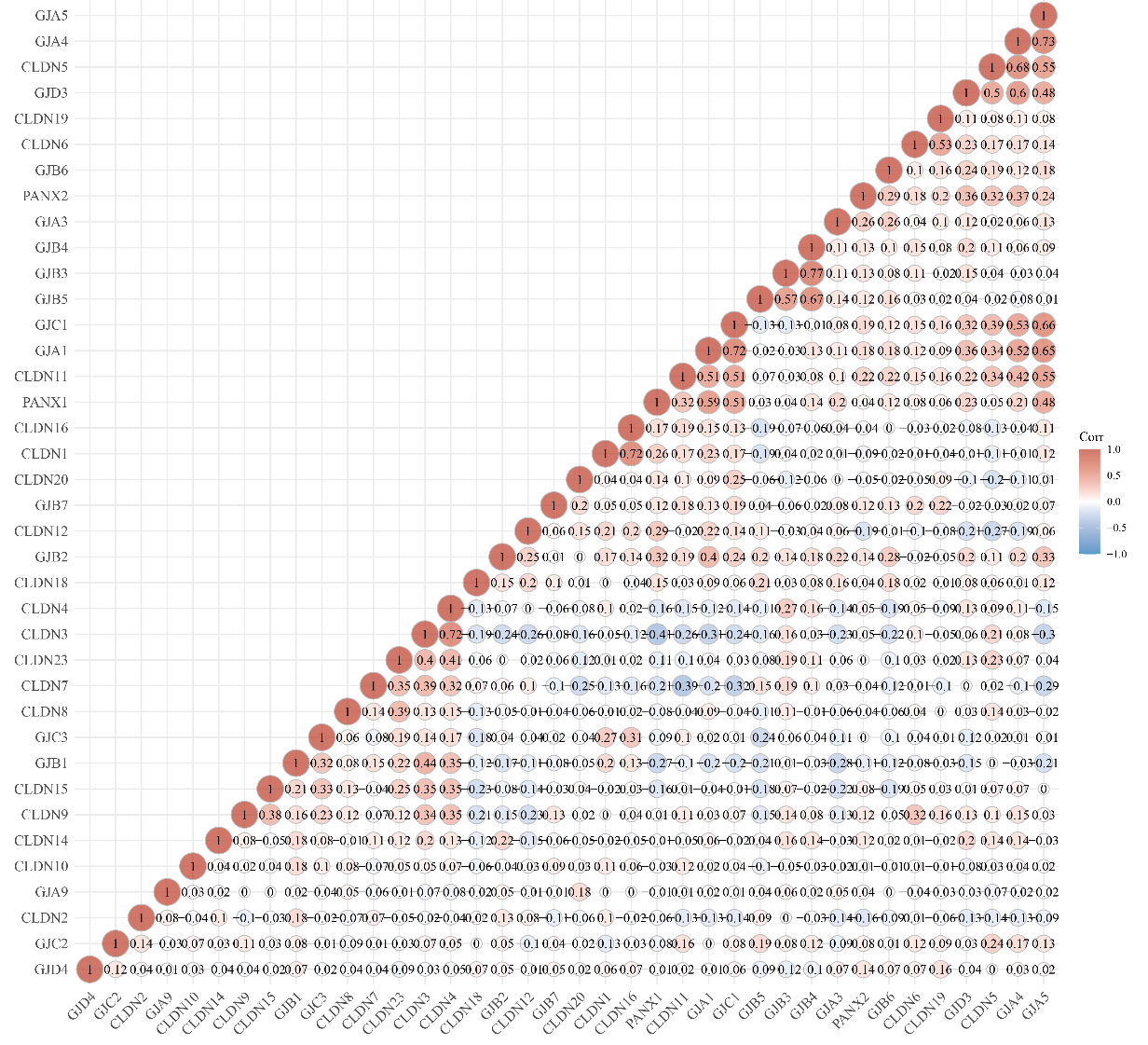


**Figure S4. correlation analysis of cell communication (CC).**

Correlation between expression levels of 47 cell communication (CC) molecules. The color of the circle represents the high and low correlation red represents positive correlation; blue represents negative correlation. (Spearman method, TCGA-CRC, n = 620).

### Figure S5

**
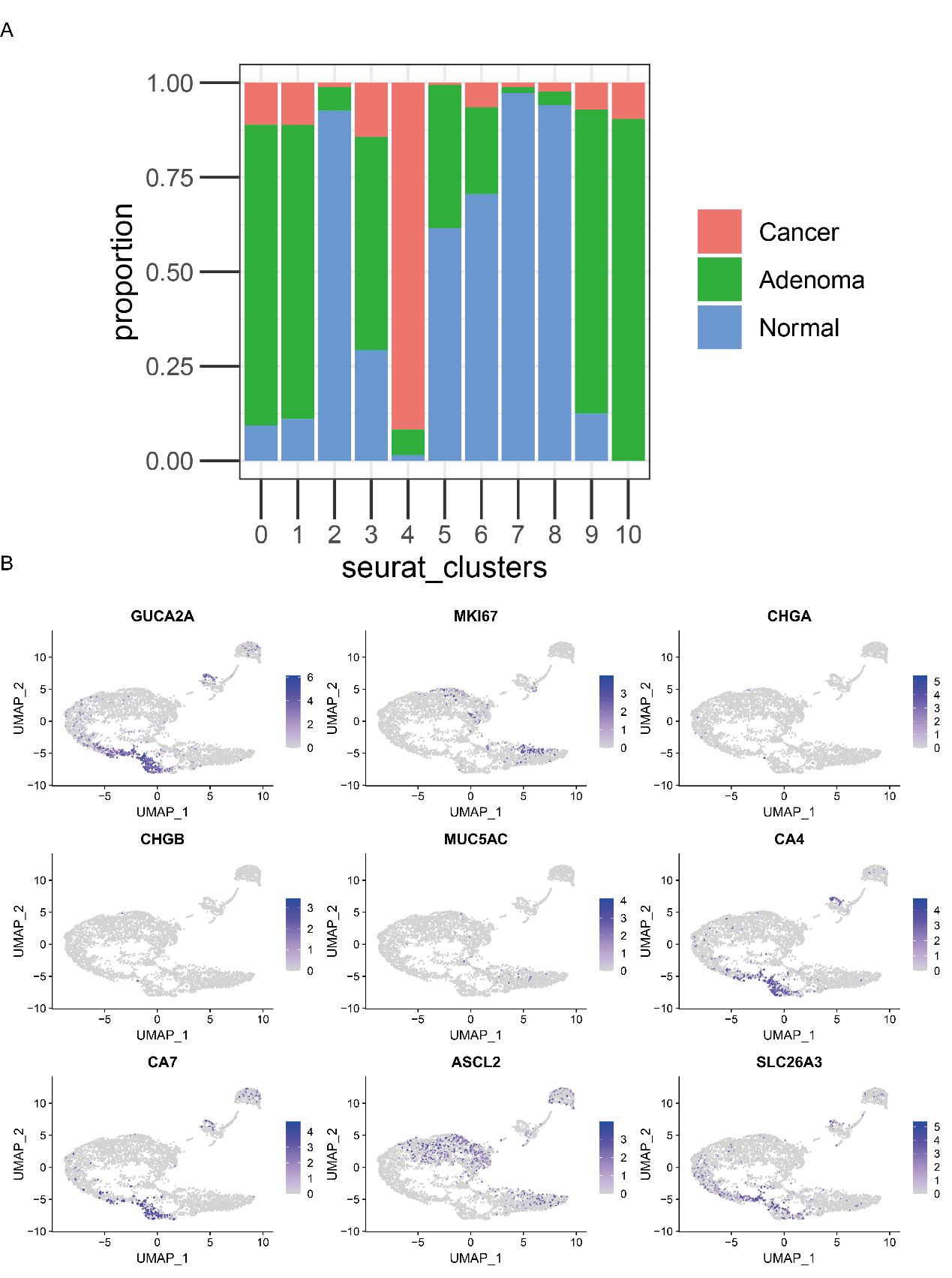
**

**Figure S5. The expression levels of well-known epithelial cell markers.**

(A) The percentage of sample origins (Cancer, Adenoma, Normal) in different seurat clusters. (B) UMAP plot show the marker genes for normal intestinal epithelial cells.

### Figure S6


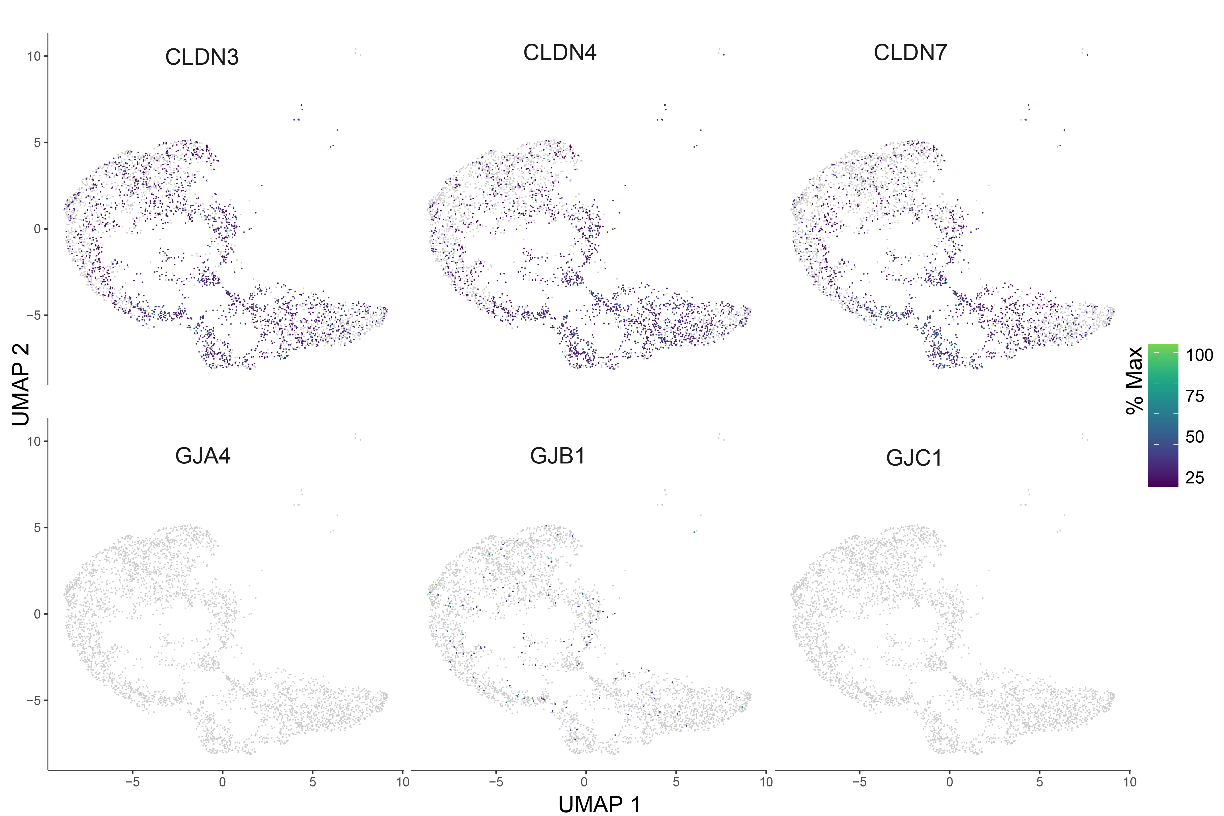


**Figure S6. The expression levels of TJs and GJs from normal to adenoma.**

UMAP dimensionality reduction visualizes the expression profiles of TJs and GJs in normal-adenoma transdifferentiation (CLDN3, CLDN4, and CLDN7 to represent TJs and GJA4, GJB1, and GJC1 to represent GJs).

### Figure S7


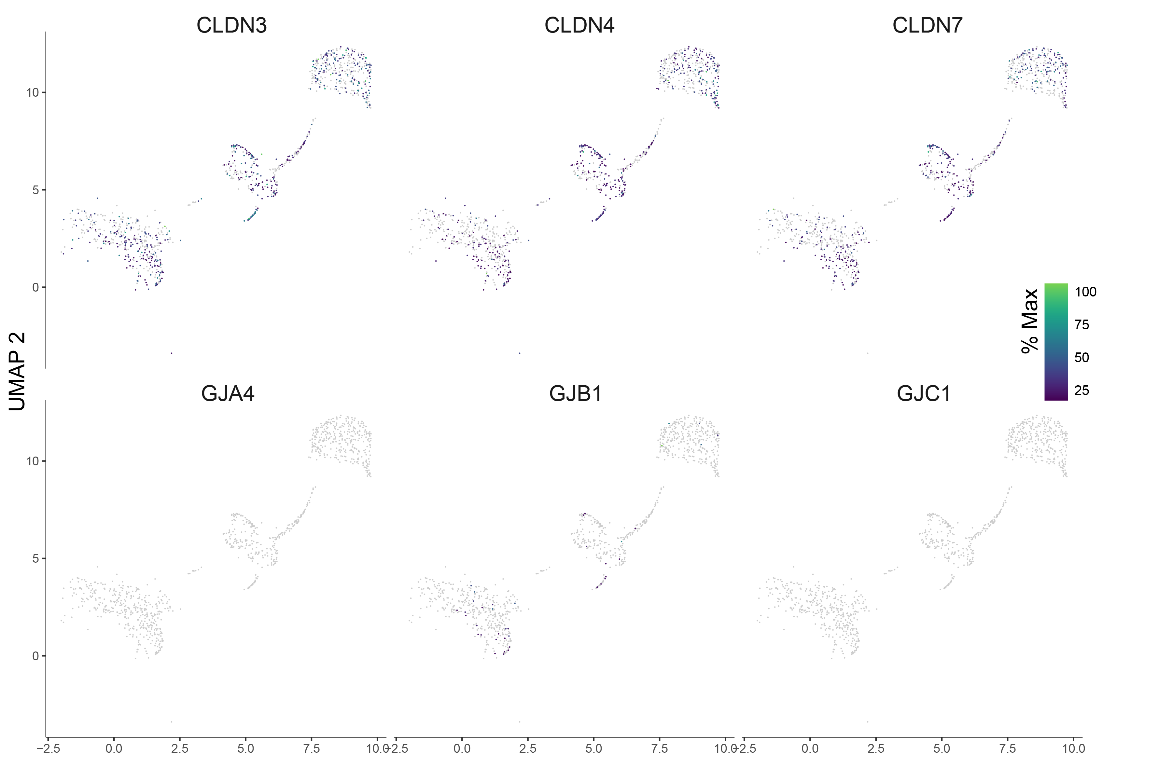


**Figure S7.** **The expression levels of TJs and GJs from normal to cancer.**

UMAP dimensionality reduction visualizes the expression profiles of TJs and GJs in normal-cancer transdifferentiation.

### Figure S8


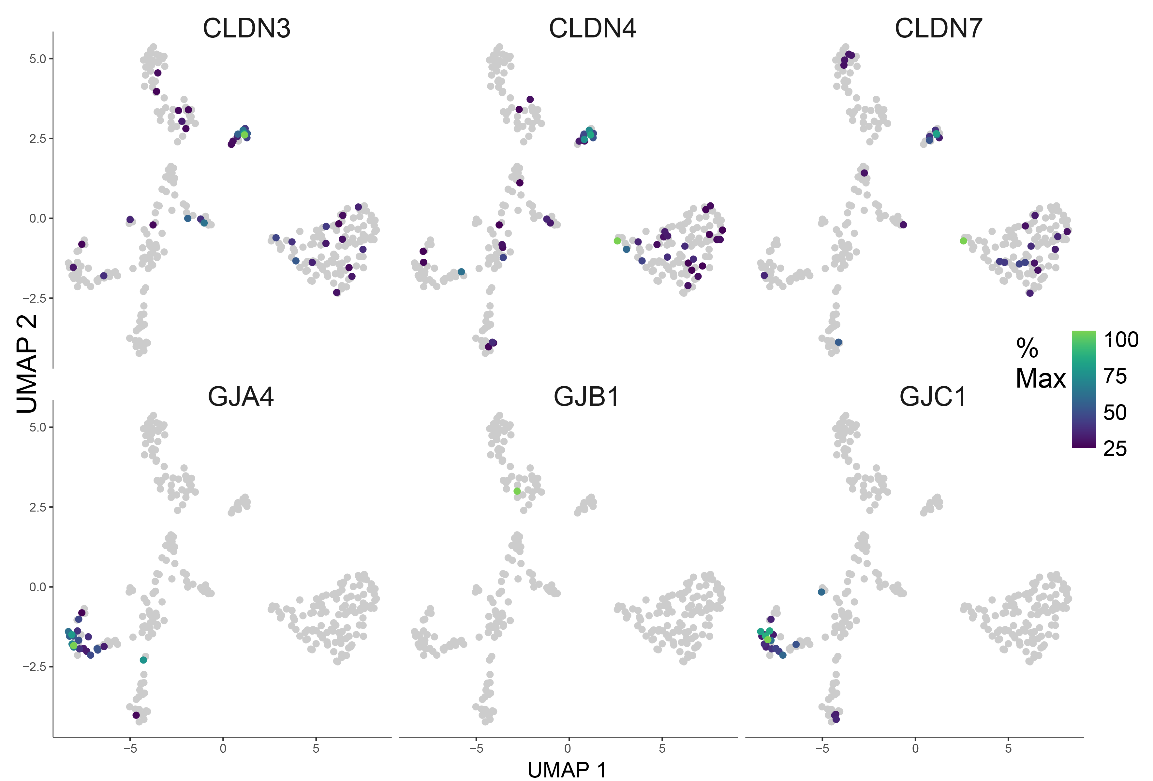


**Figure S8.** **The expression levels of TJs and GJs from NFs to CAFs.**

UMAP dimensionality reduction visualizes the expression profiles of TJs and GJs in NFs to CAFs.

### Figure S9


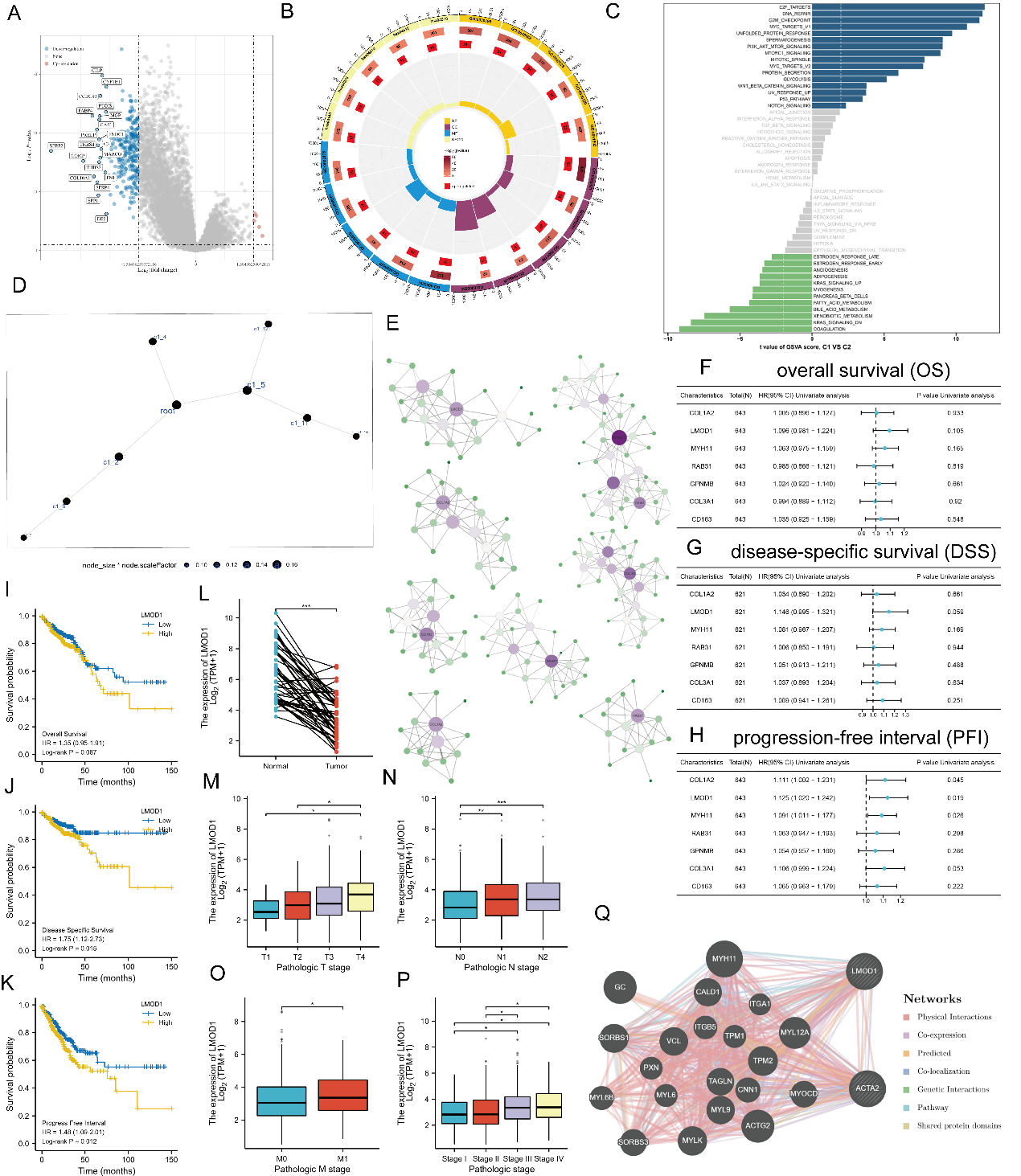


**Figure S9. Identification of LMOD1 as a potential regulator under the CC phenotype.**

(A) Volcano plot of Differentially expressed genes (DEGs) mediated by the changes in CC molecules levels. Grey dots = not statistically significant; Red dots = up-regulated in C1 and Blue dots = up-regulated in C2, with statistical significance, ordinate = the significance of differences (−log10 P value); abscissa = expression differences (log2 fold change).

(B) GO and KEGG enrichment analyses to identify specific biological pathways in two CC phenotypes (See also Table S4).

(C) GSVA enrichment analysis shows the activation status of biological pathways between 2 different Cell Communication subtypes. Bar plot were used to visualize HALLMARK geneset, with blue representing activation and green representing inhibition in C1. Gray regions stand for not statistically significant.

(D) Multiscale Embedded Gene Co-Expression Network Analysis (MEGENA) based on co-expression network. Each node = module, where larger nodes = an increased number of genes.

(E)The MEGENA network shows the top 8 gene modules. The larger the size, the purpler the colour, the more important it is in the network.

(F-H) The forest map shows the results of Cox regression analysis on the average survival rate of 7 hub genes from MEGENA modules in the TCGA-CRC cohort, including OS (F), DSS (G), and PFI (H).

(I-K) Kaplan-Meier curves of patients’ OS (I), DSS (J), and PFI (K) prediction are plotted based on LMOD1 levels in TCGA-CRC.

(L) The box plots of LMOD1 expression between different tissue types were showed (paired normal and tumor tissues). Wilcoxon test was conducted.

(M-P) The box plots of LMOD1 expression across different clinical stages were showed, including T (M), N (N), M (O), and pathological stages (P). Kruskal-Wallis test was conducted.

(Q) The gene–gene interaction network for *LMOD1* and neighboring genes was analyzed using the GeneMANIA database. Each node represents a gene. The line color represents possible relationship between the respective genes.

### Figure S10


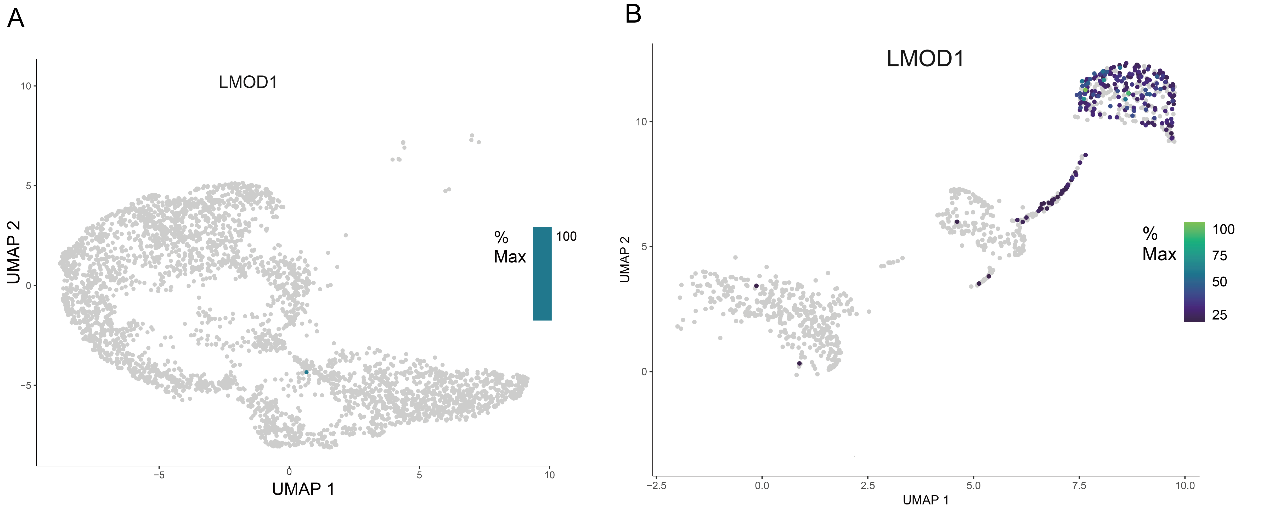


**Figure S10.** **The expression levels of *LMOD1* in two transdifferentiation pathways.**

UMAP dimensionality reduction visualizes the expression profiles of *LMOD1* in normal-adenoma transdifferentiation (A) and normal-cancer transdifferentiation (B).

### Figure S11


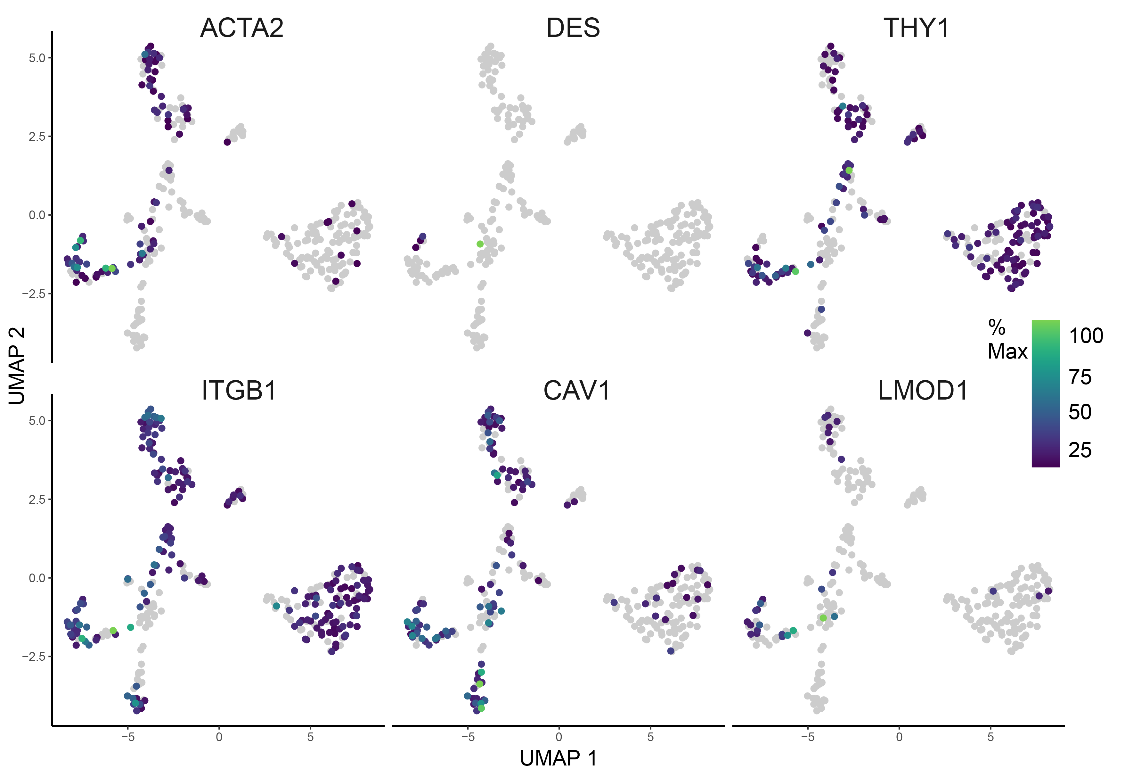


**Figure S11. The expression levels of *LMOD1* and fibroblast activation markers from NFs to CAFs.**

UMAP dimensionality reduction visualizes the similarity of expression profiles of *LMOD1* and fibroblast activation markers.

**References**

1. Pagès, F., et al., *International validation of the consensus Immunoscore for the classification of colon cancer: a prognostic and accuracy study.* Lancet, 2018. **391**(10135): p. 2128-2139.
